# BODIPY Conjugates as Functional Compounds for Medical Diagnostics and Treatment

**DOI:** 10.3390/molecules27041396

**Published:** 2022-02-18

**Authors:** Elena Antina, Natalia Bumagina, Yuriy Marfin, Galina Guseva, Liliya Nikitina, Dmitry Sbytov, Felix Telegin

**Affiliations:** 1 G.A. Krestov Institute of Solution Chemistry of Russian Academy of Sciences, 1 Akademicheskaya St., 153045 Ivanovo, Russia; eva@isc-ras.ru (E.A.); nad@isc-ras.ru (N.B.); gbg@isc-ras.ru (G.G.); 2 Inorganic Chemistry Department, Ivanovo State University of Chemistry and Technology, 7 Sheremetevskiy Ave., 153000 Ivanovo, Russia; sbytov.dima@mail.ru (D.S.); felix.telegin@gmail.com (F.T.); 3 Department of General and Organic Chemistry, Kazan State Medical University, 49 Butlerova St., 420012 Kazan, Russia; nikitl@mail.ru; 4 Biologically Active Terpenoids Laboratory, Kazan Federal University, 18 Kremlyovskaya St., 420008 Kazan, Russia

**Keywords:** BODIPY, conjugate, drug delivery, fluorescent label, luminophore, biomarker

## Abstract

Fluorescent dyes absorbing and emitting in the visible and near-IR regions are promising for the development of fluorescent probes for labeling and bio-visualization of body cells. The ability to absorb and emit in the long-wavelength region increases the efficiency of recording the spectral signals of the probes due to the higher permeability of the skin layers. Compared to other fluorescent dyes, BODIPYs are attractive due to their excellent photophysical properties–narrow absorption and emission, intense fluorescence, simple signal modulation for the practical applications. As part of conjugates with biomolecules, BODIPY could act as a biomarker, but as therapeutic agent, which allows solving several problems at once-labeling or bioimaging and treatment based on the suppression of pathogenic microflora and cancer cells, which provides a huge potential for practical application of BODIPY conjugates in medicine. The review is devoted to the discussion of the recent, promising directions of BODIPY application in the field of conjugation with biomolecules. The first direction is associated with the development of BODIPY conjugates with drugs, including compounds of platinum, paclitaxel, chlorambucil, isoxazole, capsaicin, etc. The second direction is devoted to the labeling of vitamins, hormones, lipids, and other biomolecules to control the processes of their transport, localization in target cells, and metabolism. Within the framework of the third direction, the problem of obtaining functional optically active materials by conjugating BODIPY with other colored and fluorescent particles, in particular, phthalocyanines, is being solved.

## 1. Introduction

Currently, research on the development and creation of theranostic drugs, that can perform two or more functions in living organisms: a colorimetric or fluorescent imager, marker, sensor, diagnostic, therapeutic, or transport agent are actively developed. For example, bio-conjugates based on the molecules of bioactive compound and luminophor are one of the promising anticancer drugs. The luminophor provides visual control over the processes of target-delivery, selective accumulation, and localization of a drug compound in cells. Bio-conjugate, as a rule, contains a luminophor, bound to a drug or other biocompound via a linker, which can be cleaved under physiological conditions, releasing the active form of the drug. The luminophor itself can also perform the functions of a drug, for example, taking part in the destruction of cancer cells, acting as a PDT agent of I or II types. This increases the effectiveness of an antitumor drug based on bio-conjugates in vitro and in vivo. Such multifunctional bio-conjugates must satisfy a number of basic requirements: intensely absorb and/or emit in the region of the phototherapeutic window, generate singlet oxygen, be localized predominantly in target cells, including cancer cells, as compared to “normal” healthy cells, have a low level of cytotoxicity for healthy cells compared to cancer cells.

Note, that numerous studies confirm the low level of cytotoxicity of many bio-conjugates for healthy cells as compared to cancer cells. This may be due to the fact that such compounds are predominantly localized in lysosomes. Cancer cells contain more lysosomes than normal cells, and therefore, by targeting drugs to the lysosomes, significant side effects of the drug can be reduced.

**Figure 1:**
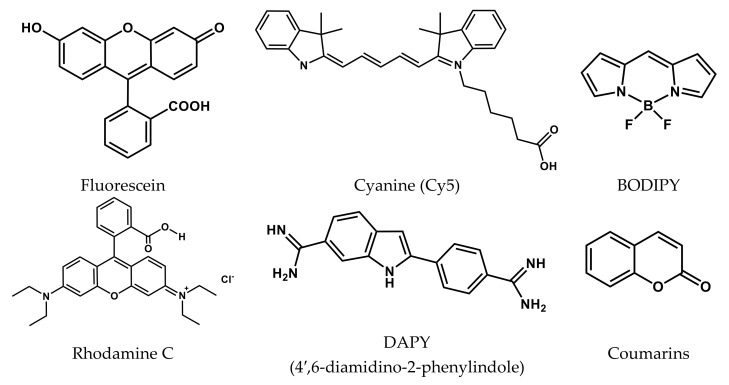
Structures of organic dyes.

The cyanine, rhodamine, fluorescein, and other dyes, among which boron(III) dipyrromethenates (BODIPYs), are the most promising organic dyes for bioapplications, can act as luminophors in the composition of bio-conjugates (Figure 1).

Via simple structural modification, one can easily control the most important properties of BODIPYs derivatives: high extinction coefficients and intense fluorescence in the visible and NIR spectral regions, high photo- and thermal stability. Due to their low cytotoxicity, alkyl-substituted BODIPYs are ideal agents for cell imaging and sensing. The low photocytotoxicity of halogen-substituted BODIPYs for healthy cells is due to their selective localization in cancer cells. Along with this, absorption and luminescence in the visible and NIR ranges (400–720 nm) and high values of the quantum yield of singlet oxygen generation along the type II pathway make them effective photosensitizers for PDT of cancer cells.

**Figure 2:**
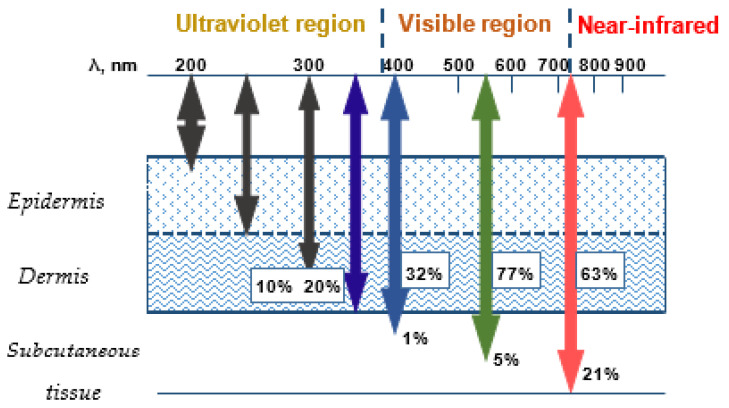
The absorption ranges.

Simple modification of the BODIPY structure allows one to obtain fluorophores with spectral characteristics in a specific spectral region, which is determined by the location of cancer cells (Figure 2). Fluorophores, that absorb and emit in the visible region of the spectrum, are able to pass into an excited state, localizing in the near subcutaneous layers, and compounds with spectral characteristics in the NIR region of the “phototherapeutic window” are capable of singlet oxygen generation in the deeper layers of the subcutaneous tissue.

For conjugation with vitamins, peptides, steroids, lipids, drugs, and other biologically active compounds, BODIPY molecules must contain a functional group (hydroclyl, carboxyl, formyl, pyridyl, etc.) in the composition of meso- or pyrrole substituents. The resulting BODIPY bio-conjugates, as a rule, have very individual properties, specifically manifesting themselves in medical and biochemical research. The high specificity of the properties of individual conjugates is determined by both the nature of the components and the method of their conjugation. The review discusses the main examples of using BODIPY bio-conjugates with drugs, vitamins, hormones, steroids, lipids, and some other bioactive compounds, demonstrating broad prospects for using BODIPY conjugates in various fields of medicine and biochemistry.

## 2. Multifunctional Bio-Conjugates BODIPY for Bioimaging and Therapy

To date, the direction of obtaining and studying BODIPY bio-conjugates, which perform the functions of theranostic agents in cancer therapy, is the most developed. Much less results were obtained for BODIPY bio-conjugates, which suppress the development of pathogenic microorganisms (bacteria, fungi, viruses, etc.).

### 2.1. BODIPY Conjugates with Platinum Drugs

Currently, Pt(II) complexes, informally called simply “platinum”, are used as cytostatic antitumor chemotherapeutic drugs [1,2,3,4,5]. Among them, cisplatin is widely known as a first-generation drug and its analogs—carboplatin and oxoplatin—are of the second and third generation. However, the non-fluorescent nature of platinum-based drugs becomes an obstacle to tracking the processes of translocation, drug release, and excretion of anticancer agents. The introduction of BODIPY-luminophor into antitumor Pt drugs allows cancer therapy under the control of fluorescence imaging. In this section, for convenience, Table 1—with the main parameters for the studied conjugates presented in the cited articles—was added.

**Scheme 1:**
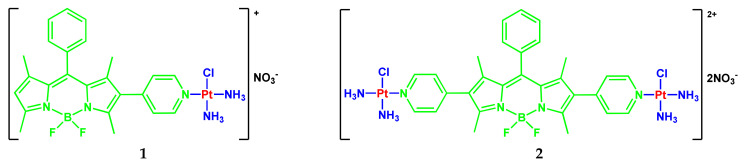
Structures of compounds **1** and **2**.

The authors of [6] developed two BODIPY-based monofunctional Pt(II) complexes **1** and **2** for PDT (Scheme 1).

The photophysical properties of BODIPY–Pt complexes were studied. Mono- and bi-substituted conjugates **1** and **2** with platinum complexes are characterized by intense absorption in the range of 506–525 nm with extinction coefficients 26,300–78,300 M^−1^cm^−1^. The absolute fluorescence quantum yield of such conjugates reaches 55% in DMSO.

Conjugates **1** and **2** generate singlet oxygen with quantum yields are 0.19 and 0.14, respectively, which makes them promising for use in photodynamic therapy. Both conjugates showed the ability to selectively suppress tumor development after light irradiation. For **1** under irradiation, the IC_50_ against of the cancer cell lines was 13.1 μM for HeLa cells and 7.6 μM for MCF-7 cells, while the IC_50_ values for normal cell lines were 32.4 μM for HBL-100 cells and 48.6 μM for L02 cells. Compound **1** exhibits high specific (selective) phototoxicity towards cancer cells.

**Scheme 2:**
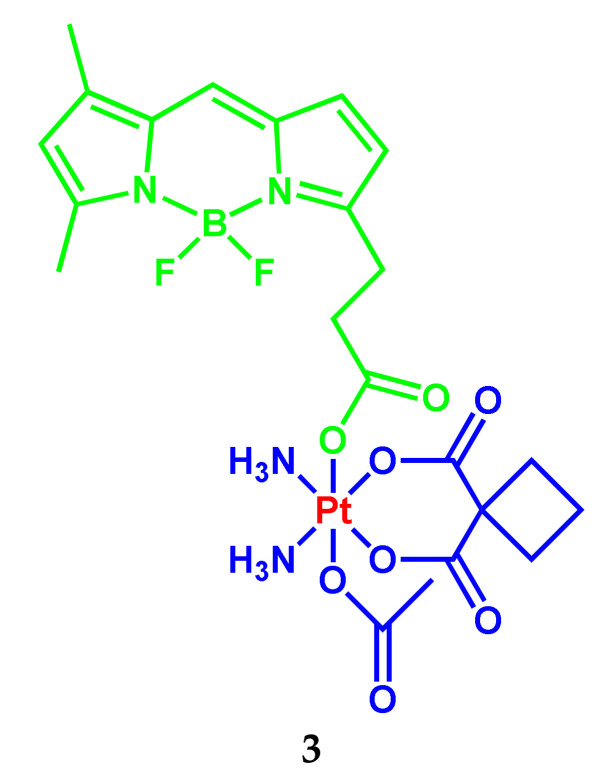
Structure of compound **3**.

The BODIPY-Pt(IV) conjugate 3 with increased cytotoxicity against cancer cells compared to carboplatin was developed (Scheme 2) [7].

The chromophore characteristics of **3** practically do not change in comparison with the unconjugated dye. However, the fluorescence quantum yield of the conjugate **3** decreases by more than an order of magnitude: from 0.81 for unconjugated BODIPY fluorophore to 0.03 for the BODIPY-Pt(IV) conjugate **3**. In this case, the efficiency of the singlet oxygen generation upon irradiation of the luminophor in the composition of **3** is noticeably lower compared to unconjugated BODIPY, probably due to the loss of the energy part of the ligand for the reduction of platinum(IV). The resulting BODIPY-Pt(IV) **3** is quite stable in PBS buffer even in the presence of reducing agents, but it can be rapidly activated under green light to form BODIPY and carboplatin. This pro-drug exhibits increased photocytotoxicity against cancer cells compared to carboplatin and is not cytotoxic against normal cells in the dark. The increased photocytotoxicity is partly due to increased cell accumulation and the subsequent increase in binding Pt in genomic DNA.

**Scheme 3:**
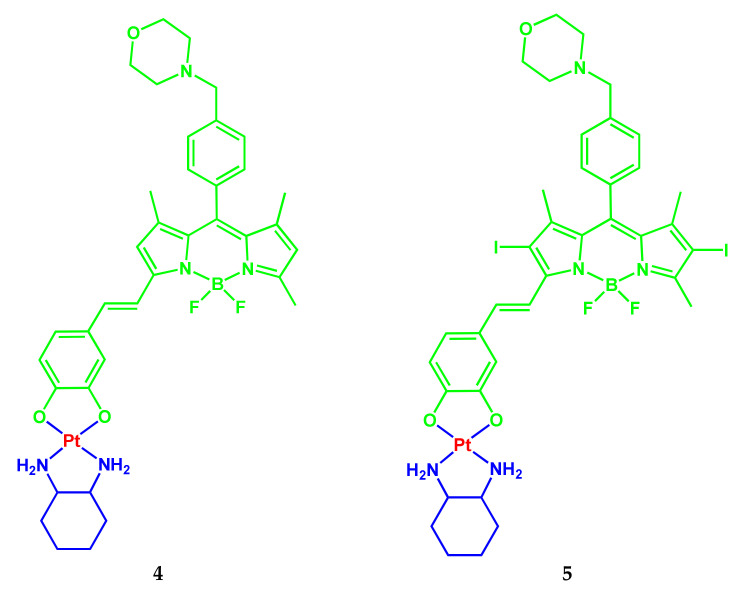
Structures of compounds **4** and **5**.

BODIPY-platinum(II) complexes **4** and **5** based on the O,O-donor monostyryl-substituted BODIPYs were developed [8] for photodynamic therapy in NIR light (600–720 nm) by targeting lysosomes (Scheme 3).

The maximum absorption bands of compounds **4** and **5** are in the PDT spectral window at the 596 and 710 nm, respectively, with high absorption coefficients (2 × 10^4^ M^−1^ cm^−1^–4 × 10^4^ M^−1^ cm^−1^) in DMSO (10%)-DMEM of pH 7.2. The conjugates **4** and **5** exhibit weak fluorescence with a maximum of the fluorescence bands of non-iodinated and diiodinated conjugates at 615 and 718 nm, respectively. Low values of the fluorescence quantum yield of conjugates (for non-iodinated conjugate 4%) limit the possibility of bioimaging studies. Both complexes are stable in the dark for up to 48 h. When irradiated with visible light (400–700 nm), the non-iodinated conjugate **4** was found to be stable, while the conjugate **5** with diiodinated BODIPY begins to decompose within 1 h after exposure to light (400–700 nm), possibly due to the presence of iodine atoms in the BODIPY nucleus.

The IC_50_ values for diiodinated conjugate **5** under red light (λ 600–720 nm; light dose 30 J/cm^2^) were 0.8 and 1.19 μM in HeLa and A549 cancer cells, respectively, which is 10 times lower than the corresponding unconjugated BODIPY. A similar trend was observed in the IC_50_ values for the non-iodinated conjugate **4**. In contrast to IC_50_ values in lung adenocarcinoma cells A549, the IC_50_ values in HPL1D cells are about 30 times higher for iodinated conjugate **5** and about 10 times higher for non-iodinated conjugate **4**, indicating their lower toxicity to normal cells.

The results, obtained by the authors in [8] from the cytotoxicity analysis showed that the diiodinated conjugate **5** can be entered to kill cancer cells when irradiated with red light without causing any significant damage to non-cancerous cells. Due to intense absorption bands, these complexes are suitable for both cellular tracking/imaging and near-IR light PDT to treat cancer cells.

**Scheme 4:**
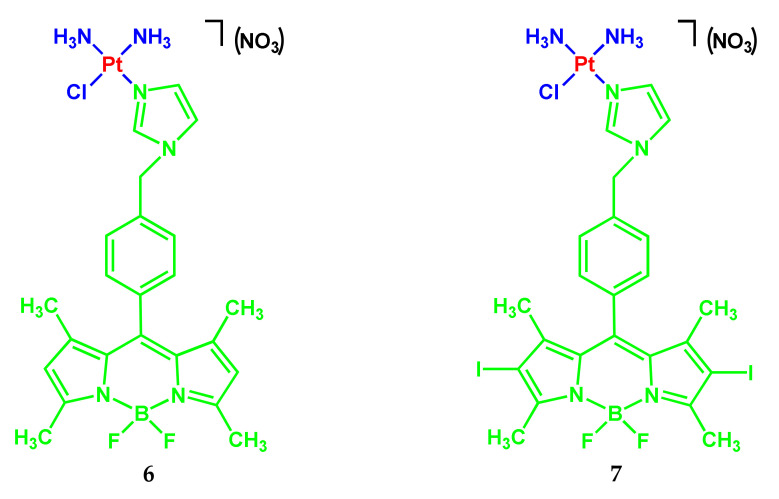
Structures of compounds **6** and **7**.

**Scheme 5:**
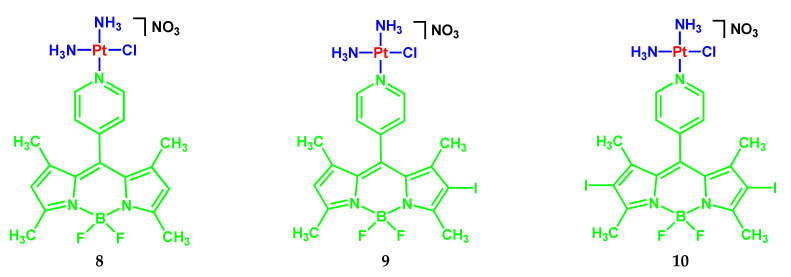
Structures of compounds **8**, **9** and **10**.

The monofunctional BODIPY conjugates with imidazoplatin **6**–**7** (Scheme 4) [9] and pyriplatin **8**–**10** (Scheme 5) [10] for cellular imaging and mitochondria-targeted photocytotoxicity were developed by a group of scientists from India.

Complexes **6** and **7** demonstrated strong absorption bands in the visible region at 500 nm (ε = 27,000 M^−1^ cm^−1^) and 510 nm (19,000 M^−1^ cm^−1^), respectively [9]. Fluorescence of complex **6** has a band at 510 nm with a quantum yield of 0.09 in 1% DMSO/DMEM (pH 7.2). Complex **7** containing the diiodo-BODIPY unit is non-emissive.

The possibility of singlet oxygen generation upon photoirradiation with visible light (400–700 nm) was shown. Research demonstrated a significant photosensitizing ability of the BODIPY complexes **6** and **7**. Both conjugates were remarkably photocytotoxic in visible light against HaCaT and MCF-7 cells. The IC_50_ values are in nanomolar concentration. The complexes were essentially nontoxic to the cells in the dark (IC_50_ > 80 μM). Complex **6** showed significant mitochondrial localization using confocal microscopy.

The authors note the effect of PDT, induced by singlet oxygen of non-fluorescent complex **7** (“Imidazoplatin-B”) with an IC_50_ value at nanomolar concentration, while visualization of cells performed using fluorescent complex **6** showed its significant mitochondrial localization in HaCaT and MCF-7 cells.

Monofunctional analogs of pyriplatin **8**–**10** containing BODIPY with meso-pyridyl fragments: intensely fluorescent BODIPY **8**, moderately fluorescent monoiodine-BODIPY **9**, and non-fluorescent diiodine-BODIPY **10** were developed as platinum-based PDT agents (Scheme 5) [10].

The Pt-BODIPY conjugates **8**–**10** showed absorption with a band maximum in the range of 505–550 nm and a green fluorescence band at ~535 nm in 1% DMSO/DMEM buffer. Mono- and diiodinated complexes BODIPY **9** and **10** as effective photosensitizers showed the singlet oxygen generation upon light activation. The ΦΔ values decreases in the following order: **10** (0.65) > **9** (0.48) > **8** (0.26).

The cytotoxicity of BODIPY conjugates **8**–**10** was studied against A549, MCF-7, and HaCaT in the dark and in visible light. The IC_50_ values range from 0.05–0.13 μM for complexes **9** and **10**, which indicate excellent photocytotoxicity; however, they remained practically non-toxic in the dark (IC_50_ > 100 μM).

Fluorescent complexes **8** and **9** with unsubstituted and monoiodosubstituted beta-positions of pyrroles in BODIPY were used for cellular imaging. Mitochondrial localization of complexes **8** and **9** was studied using confocal microscopy. Photo-mediated apoptotic cell death was confirmed using flow cytometric assays, annexin-V/FITC-PI (fluorescein isothiocyanate-propidium iodide), and cell arrest in sub-G1 and G2/M phases. The combination of photodynamic therapy with DNA crosslinking enhances the anticancer potential of monofunctional BODIPY conjugates of pyriplatines. It was shown that complex **8** is primarily suitable for the cellular imaging study with its high Φ_F_ value; complex **10** is a potential PDT agent with a high ΦΔ value. Interestingly, complex **9** with the monoiodo-BODY nucleus is suitable for both cellular imaging and PDT activity with moderate Φ_F_ and ΦΔ values.

**Scheme 6:**
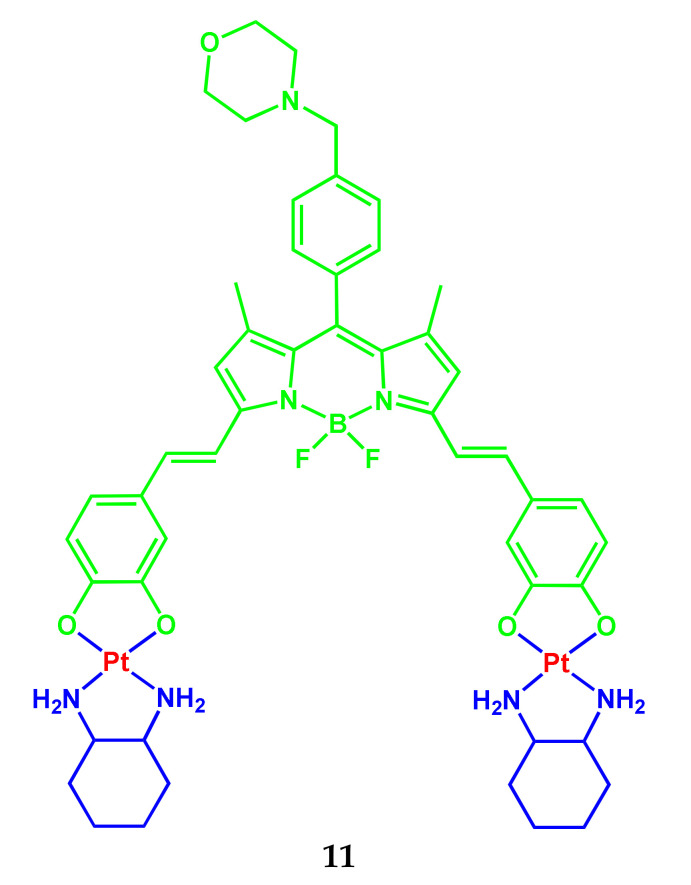
Structure of compound **11**.

Another example of a conjugate **11** based on BODIPY and cisplatin (Scheme 6) was presented in [11] by the same group of scientists from India.

The conjugate **11** is stable for up to 48 h in the dark and exposed to visible light (400–700 nm). The electronic absorption spectrum of complex **11** in 10% DMSO-DMEM (pH 7.2) is characterized by bands corresponding to electronic transitions with the participation of the distyryl BODIPY unit in the range 650–660 nm and shoulder at ~595 nm. The emission spectrum of the conjugate **11** contains a maximum at 668 nm (λ_ex_ 650 nm). The conjugate showed red fluorescence in DMSO with a quantum yield of 2%. Complex **11** has a lower fluorescence quantum yield compared to unconjugated BODIPY, which indicates an increase in the photosensitizing ability of the BODIPY fragment upon binding to the platinum(II) complex. 

The IC_50_ values for **11** ranged from 0.6–3.4 μM in cancer cells in red light (600–720 nm) with insignificant cytotoxicity in the dark. The IC_50_ values of the conjugate **11** in HeLa, A549, and MDA-MB-231 cells were 0.6 μM, 0.7 μM, and 3.4 μM, respectively. In the normal cell line HPL1D, the IC_50_ is 62 μM. In the dark with 4 h preincubation and then 20 h postincubation IC_50_ ~100 μM. The higher photocytotoxicity of **11** may be due to its higher ability to generate singlet oxygen compared to BODIPY, which does not contain the platinum complex. 

Analysis of cell localization showed that the conjugate **11** is predominantly localized in the cytoplasm of cells. Thus, in the opinion of the authors of [11], the conjugate **11** is a means of PDT in the NIR range, specifically aimed at the destruction of lysosomes of cancer cells.

**Scheme 7:**
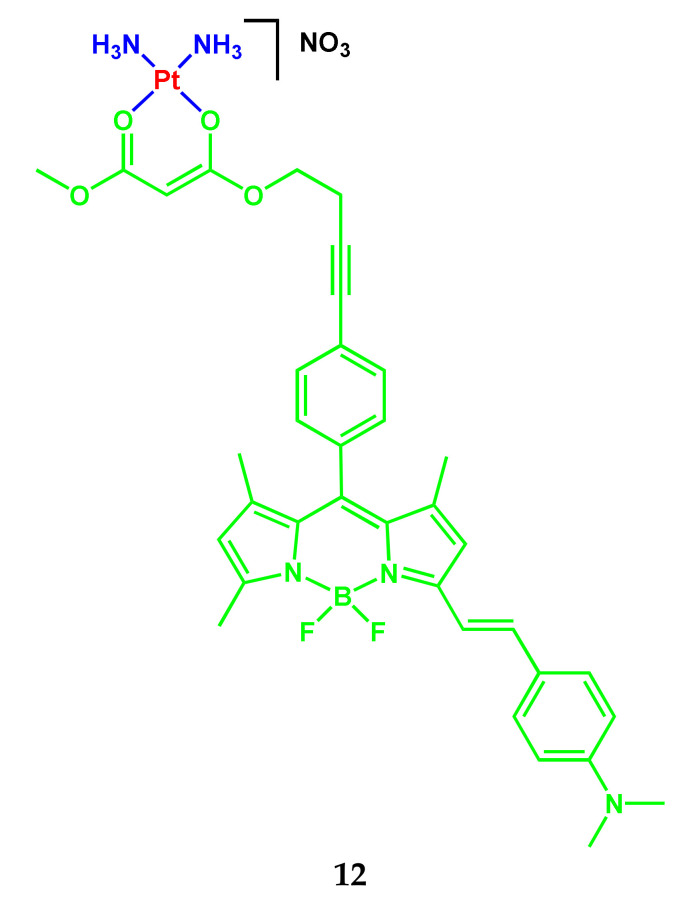
Structure of compound **12**.

A conjugate **12** based on monoaminostyryl-substituted BODIPY (Scheme 7) was presented by the authors of the article [12].

The conjugate is characterized by absorption bands at 617 nm (ε = 2.9 × 10^4^ M^−1^ cm^−1^) in pH 7.2 10% DMSO/DMEM buffer (*v*/*v*). The high value of the absorption coefficients within the PDT spectral window is conducive to a desirable photosensitizing effect. The presence of platinum as a heavy metal in **12** makes this complex suitable as a photosensitizer for PDT activity. The **12** upon excitation at 615 nm displayed a broad fluorescence band within 650−850 nm with a maximum at 720 nm. A partial charge transfer effect observed in monoaminostyryl-BODIPY leads to large Stokes shifts in the fluorescence spectra of **12**. The Φ_F_ value for **12** is 0.032. The **12** generated singlet oxygen under red-light irradiation with a ΦΔ = 0.48.

Complex **12** containing a monostyryl BODIPY moiety displayed remarkable NIR light (λ = 600−720 nm) photocytotoxicity with IC_50_ values of 1.6 and 2.4 μM in the A549 and HeLa cancer cells, respectively, with low dark cytotoxicity (IC_50_ = 85 and 79 μM, respectively). 

The possibility of any interaction of complex **12** with DNA was studied by UV−Vis absorption spectral studies using ct-DNA. The ct-DNA binding constant (K_b_) for **12** was found to be ∼3.9 × 10^6^ M^−1^ in 5% DMF-Tris buffer (pH 7.2). This means that complex **12** interacts with DNA through groove binding and/or an intercalative mode. The authors’ results of [12] showed that **12** is capable of singlet oxygen generation and exhibits a remarkable PDT effect along with a DNA binding interaction with the possibility of inducing a chemotherapeutic effect. 

**Scheme 8:**
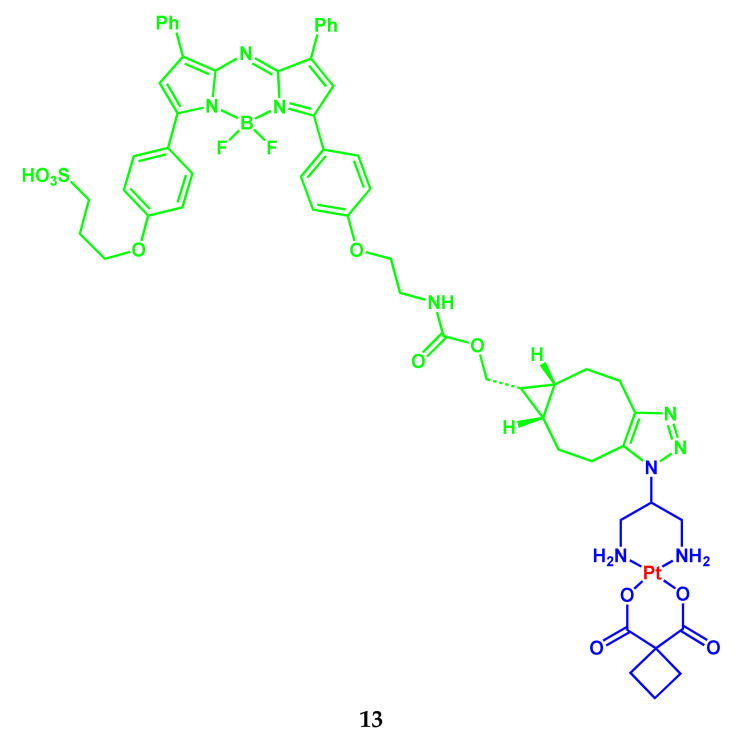
Structure of compound **13**.

Eolann Kitteringham and colleagues synthesized non-toxic carboplatin-like NIR-fluorophore conjugate **13** from cis-[Pt(2-azidopropane-1,3-diamine)(cbdca)] and bicyclo[6.1.0]non-4-yne BF_2_-azadipyrromethene fluorophore (Scheme 8) [13].

The band maxima positions in the absorption and fluorescence spectra of compound **13** are at 695 nm (ε = 64,000 M^−1^ cm^−1^) and 720 nm (Φ_F_ 0.11) in H_2_O/1% SDS. The Pt-conjugate **13** is non-toxic against cisplatin-sensitive and cisplatin-resistant ovarian cancer cell lines A2780P and A2780cisR with IC_50_ > 200 μM, is efficiently internalized in ovarian cancer cells and is evenly dispersed throughout the cytoplasm. This observation is probably associated with the expected decrease in accumulation in the nucleus of **13** compared to carboplatin.

**Scheme 9:**
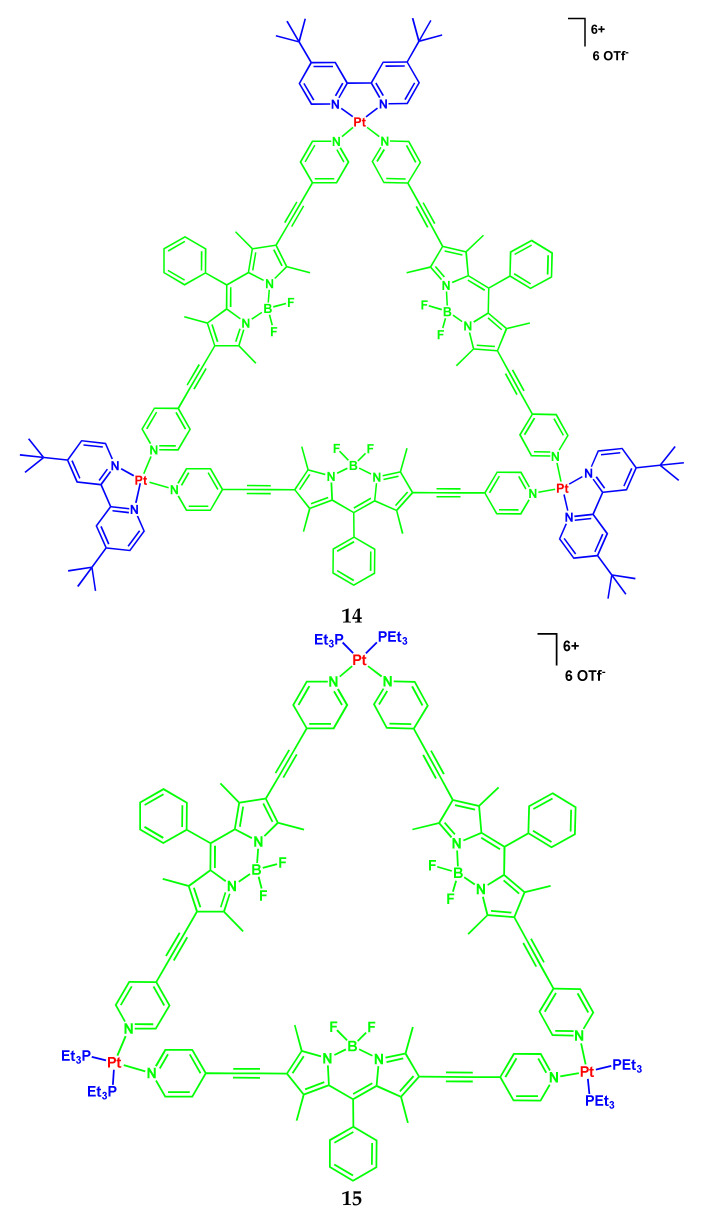
Structures of compounds **14** and **15**.

Another example of BODIPY conjugates with platinum compounds was demonstrated in the work [14]. Herein, the authors presented the coordination-driven self-assembly of two highly emissive platinum(II) supramolecular triangles **14** and **15** containing BODIPY-based bridging ligands (Scheme 9).

**Figure 3:**
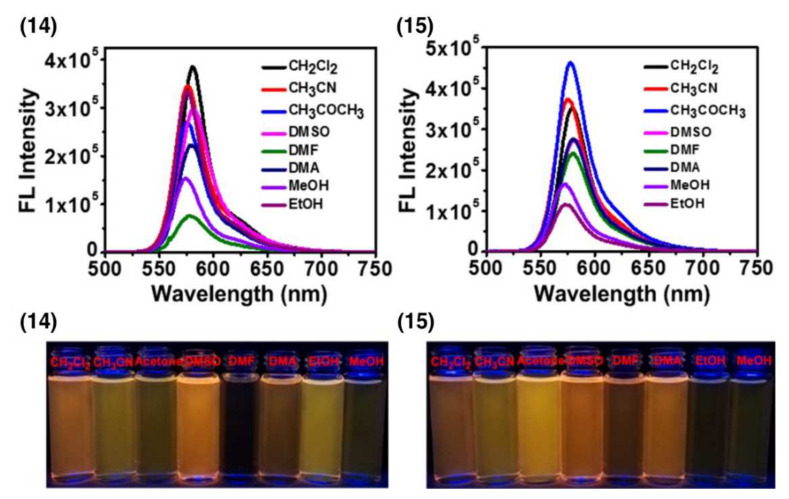
Fluorescence spectra of metallocycles **14** and **15** in different solvents (λ_ex_ = 556 nm, *c* = 1.00 μM). Photographs of **14** and **15** under UV lamp at 365 nm in different solvents. Adapted from [14].

The UV–Vis absorption spectra of the triangular metallacycles **14** and **15** showed similar absorption bands at 556 nm in different organic media, which was attributed to the π−π* transition of the BODIPY (Figure 3). BODIPY-based conjugates **14** and **15** have a high fluorescence with a fluorescence peak at 571–582 nm. The values of Φ_F_ were determined from 31.6 to 72.3% in different organic solvents in contrast to water. The fluorescence quantum yield in water decreases and was 6.9 and 13.5% for conjugates **14** and **15**, respectively. The possibility of singlet oxygen generation by metal cycles **14** and **15** was investigated.

To confirm the internalization of nanoparticles containing the metallocycles **14** or **15** in HeLa cells, studies were performed using confocal laser scanning microscopy and flow cytometry. HeLa cells exhibited strong intracellular fluorescence after incubation for 8 h with nanoparticles containing metallocycles **14** or **15**. The amount of nanoparticles containing conjugates **14** or **15** internalized by HeLa cells was determined. The study of the photocytotoxicity of metallocycles **14** and **15** against HeLa cells showed IC_50_ values of 6.41 μM and 2.11 μM, respectively. The authors evaluated the synergistic anticancer effect of conjugates **14** and **15** on HeLa cells. The IC_50_ values for **14** and **15** were 0.95 and 0.37 μM, respectively. Conjugate **15** demonstrated better antitumor efficacy, possibly due to its higher cytotoxicity and greater efficiency of internalization.

### 2.2. BODIPY-Conjugates with Curcumin

Curcumin is able to kill cancer cells and stop their growth due to the fact that it inhibits the development of new blood vessels in tumors. By stimulating the self-destruction of malignant cells, curcumin has no effect on healthy cells and does not damage them. Curcumin is able to prevent prostate cancer, stop the growth of prostate tumors, protect against breast cancer, prevent melanoma, and is able to destroy already formed cancer cells. Curcumin reduces the risk of developing leukemia in children, enhances the effects of chemotherapy, and reduces the side effects of the medications used.

**Scheme 10:**
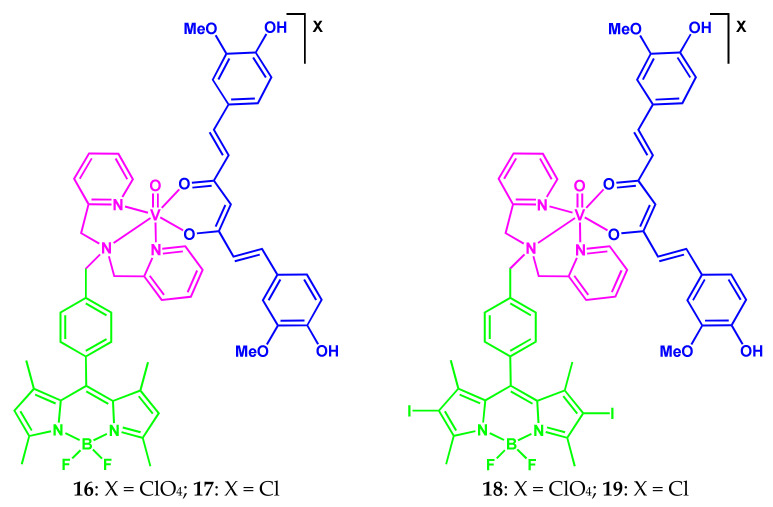
Structures of compounds **16**–**19**.

In 2017, three-component vanadium(IV) oxide conjugates **16**–**19** of curcumin, dipicolylamine containing non-iodinated or iodinated BODIPYs as side photoactive parts (Scheme 10) were presented [15].

The absorption of the most intense band is at 501 and 535 nm for complexes **16** and **18**, respectively, in DMF/PBS 1:1 (*v*/*v*) buffer. Complex **18** exhibits weak fluorescence with a quantum yield of 0.01 in 10% aqueous DMSO (maximum position at 521 nm). In contrast, non-iodinated complex **16** is characterized by significantly more intense fluorescence with a band maximum at 512 nm and a quantum yield of 0.11. Fluorescence of **16** makes it suitable for imaging studies and cellular uptake by confocal fluorescence microscopy. The conjugates are stable both in the solid phase and in solution in DMF/DPBS (1:1) up to 48 h in the dark. The non-iodinated complex **16** was found to be more stable, than the diiodinated conjugate **18**.

**Figure 4:**
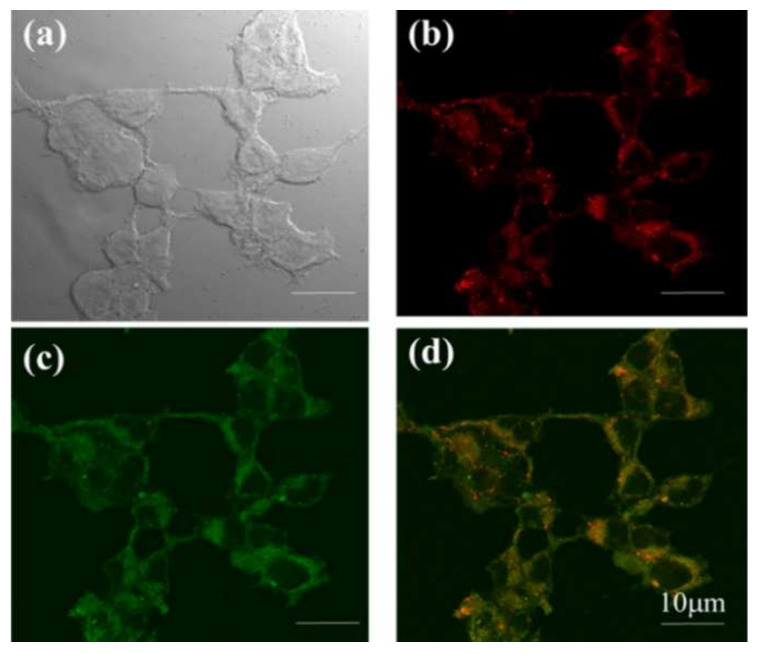
Confocal microscopic images of MCF-7 cells after 4 h of incubation with **17**: (**a**) bright field, (**b**) fluorescence of Mito Tracker Deep Red, (**c**) fluorescence of **17**, and (**d**) merged image of (**b**,**c**). Scale bar = 10 μm. Adapted from [15].

Complexes **17** and **19** showed significant cellular uptake (Figure 4). The uptake by MCF-7 within 4 h was ~90%.

The conjugates show remarkable visible-light-induced (400–700 nm, 10 J/cm^2^) cytotoxicity in MCF-7 and HeLa cells and are less toxic in the dark (IC_50_ ~100 μM). The IC_50_ values for complex **17** in the light were in the range 5.8–6.2 μM, while the photocytotoxicity of **19** is excellent, giving IC_50_ values in the 2.5–2.7 μM range. This is due to the higher ability of diiodo-BODIPY to form reactive oxygen species. The authors note that the IC_50_ values of **19** are similar to the IC_50_ values for the clinically approved PDT drug Photofrin. In experiments on visualization of MCF-7 cells, predominantly mitochondrial localization was found. The interaction of conjugates with DNA was studied by UV–Vis absorption experiments. The binding constants (*K*_b_) of the complexes for ct-DNA were ∼1 × 10^5^ M^−1^ in 5% DMF−Tris buffer (pH = 7.2). Moreover, binding constants for **17** are higher than for **19**.

### 2.3. BODIPY-Conjugates with Paclitaxel

Paclitaxel, as a powerful mitotic inhibitor of plant origin, is used in the chemotherapy of malignant tumors. The mechanism of its action is associated with the influence on the process of cell division. The drug stimulates the assembly of microtubules from tubulin dimers and stabilizes them by suppressing depolymerization, which leads to disruption of the normal process of dynamic reorganization of the microtubule network, which is important for cellular functions at the stage of mitosis and interphase of the cell cycle.

A group of conjugates based on BODIPY and paclitaxel was presented in the works [16,17,18]. Paclitaxel (Taxol) is the most commonly used cytostatic anti-cancer drug of natural origin. It is included in the List of Essential Medicines of the World Health Organization and the Russian List of Essential and Essential Medicines (Medical Drug).

**Scheme 11:**
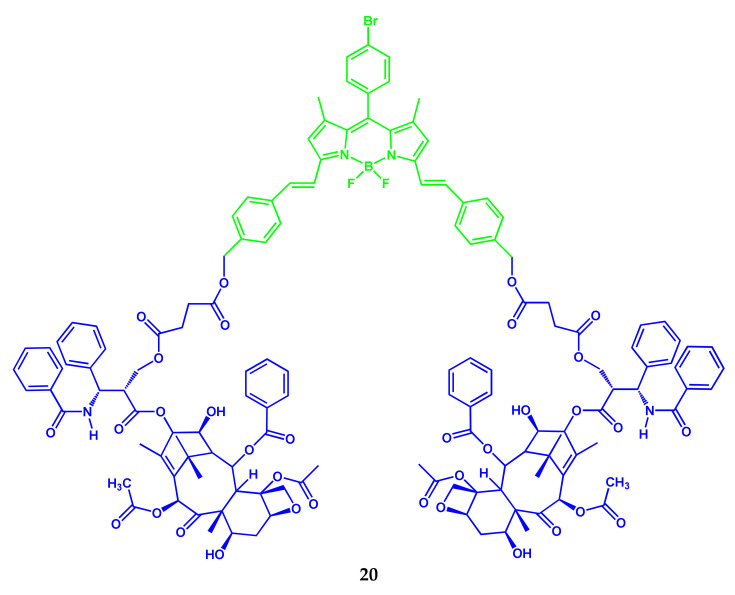
Structure of compound **20**.

An NIR-brominated BODIPY-paclitaxel conjugate **20** was synthesized by Tao Zhang and colleagues (Scheme 11) [16].

**Figure 5:**
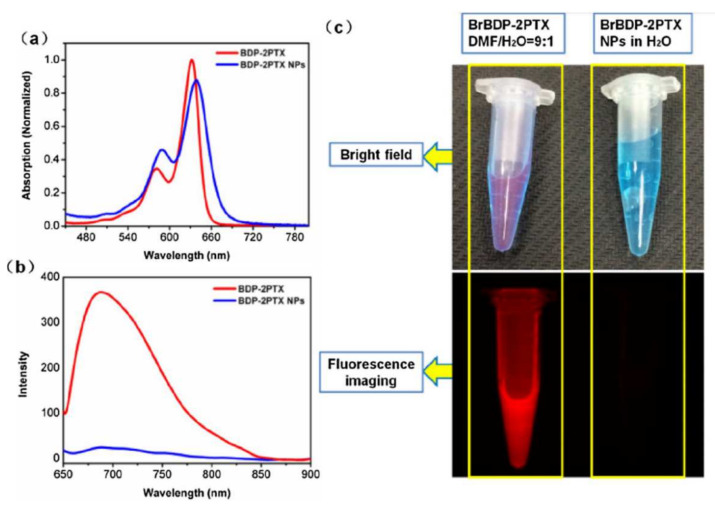
UV–Vis absorption (**a**) and fluorescence (**b**) spectra of nanoparticles **20** (NPs) in aqueous solution and free **20** in DMF/H_2_O 9:1. (**c**) Fluorescent photos **20** and NPs **20** in water and in DMF/H_2_O 9:1. Adapted from [16].

The symmetric BrBDP-2PTX **20** can be self-assembled into stable and uniform nanoparticles with a diameter of around 130 nm by the nanoprecipitation method. The maximum absorption of nanoparticles of **20** in DMF/H_2_O = 9:1 (*v*:*v*) at 639 nm was red-shifted by 7 nm relative to **20** (maximum at 632 nm) (Figure 5). This effect is indicative of J-aggregation of **20** in an aqueous solution. Relatively strong red fluorescence was observed for the conjugate (with a maximum at 688 nm), while the fluorescence of nanoparticles of **20** was quenched due to aggregation. 

The nanoparticles of **20** can release paclitaxel in living cells and exhibit effective cytotoxicity in tumor cells. Nanoparticles maintain sufficient fluorescence intensity in the tumor area for visualization and can retain it for a long time. The authors’ results of [16] indicated that the conjugate **20** could provide a potential platform for imaging-guided drug delivery and cancer treatment.

**Scheme 12:**
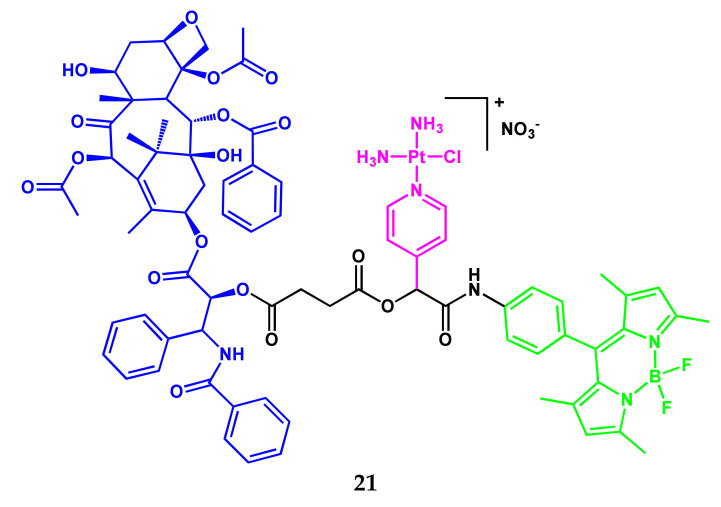
Structure of compound **21**.

A three-component amphiphilic conjugate **21** (Scheme 12) containing BODIPY-fluorophore, paclitaxel, and a platinum compound was presented in [17]. The conjugate could self-assemble into spherical nanoparticles with high stability in aqueous solution.

The optical properties of **21** in DMF/water = 9:1 (*v*/*v*) and nanoparticles of **21** in water were studied. The UV–Vis absorption spectra have the same maximum at 500.5 nm with a broader absorption peak for nanoparticles of **21**. In contrast to the more intense fluorescence of the conjugate **21** with a relative fluorescence quantum yield of 0.54, nanoparticles of **21** exhibit fluorescence with a relative Φ_F_ of less than 0.01 in water. The band maximum in the fluorescence spectrum of nanoparticles **21** was determined at 539.5 nm and shifted to the red region by 20 nm relative to the conjugate itself. The nanoparticles of **21** can be internalized by A549 and MCF-7 cancer cells (after incubation for 0.5 h) through endocytosis and simultaneously perform imaging and cytostatic functions. The results obtained are promising from the point of view of the development of nanomedical drugs based on amphiphilic conjugates of drugs with a dye for visualization of cancer cells and chemotherapy. 

**Scheme 13:**
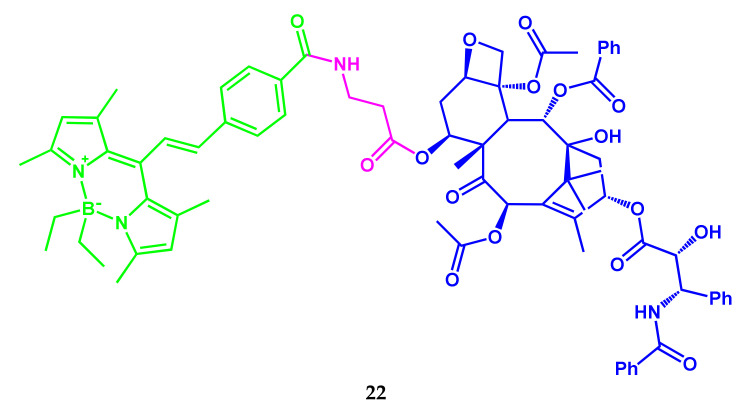
Structure of compound **22**.

In [18], the synthesis and application of a photo-activated BODIPY probe **22** with paclitaxel for single-molecule localization microscopies to obtain ultra-high-resolution images of tubulin microtubules were reported (Scheme 13).

The BODIPY-probe can be easily coupled to molecules containing nucleophiles to target specific cellular organelles or to visualize various biological targets. The authors of [18] emphasize that this is the first example of ultra-high-resolution microscopy, based on the localization of a single molecule, using the visible light-activated BODIPY as a fluorescent probe.

### 2.4. BODIPY-PEG Conjugates

One of the most important tasks is the development of BODIPY luminophor forms, that are soluble in water and physiological media, not prone to the formation of non-fluorescent aggregates, which makes them suitable for biological and medical use. The addition of hydrophilic fragments, such as polyethylene glycol, N,N-bis(2-hydroxyethyl)amine, carbohydrates, nucleotides, or ionic groups of carboxylic, sulfonic acids, or ammonium, to the BODIPY nucleus is one of the promising approaches for the synthesis of water-soluble BODIPY forms. The attachment of PEG fragments to therapeutic agents improves their water solubility, cellular uptake, and overall therapeutic efficiency.

**Scheme 14:**
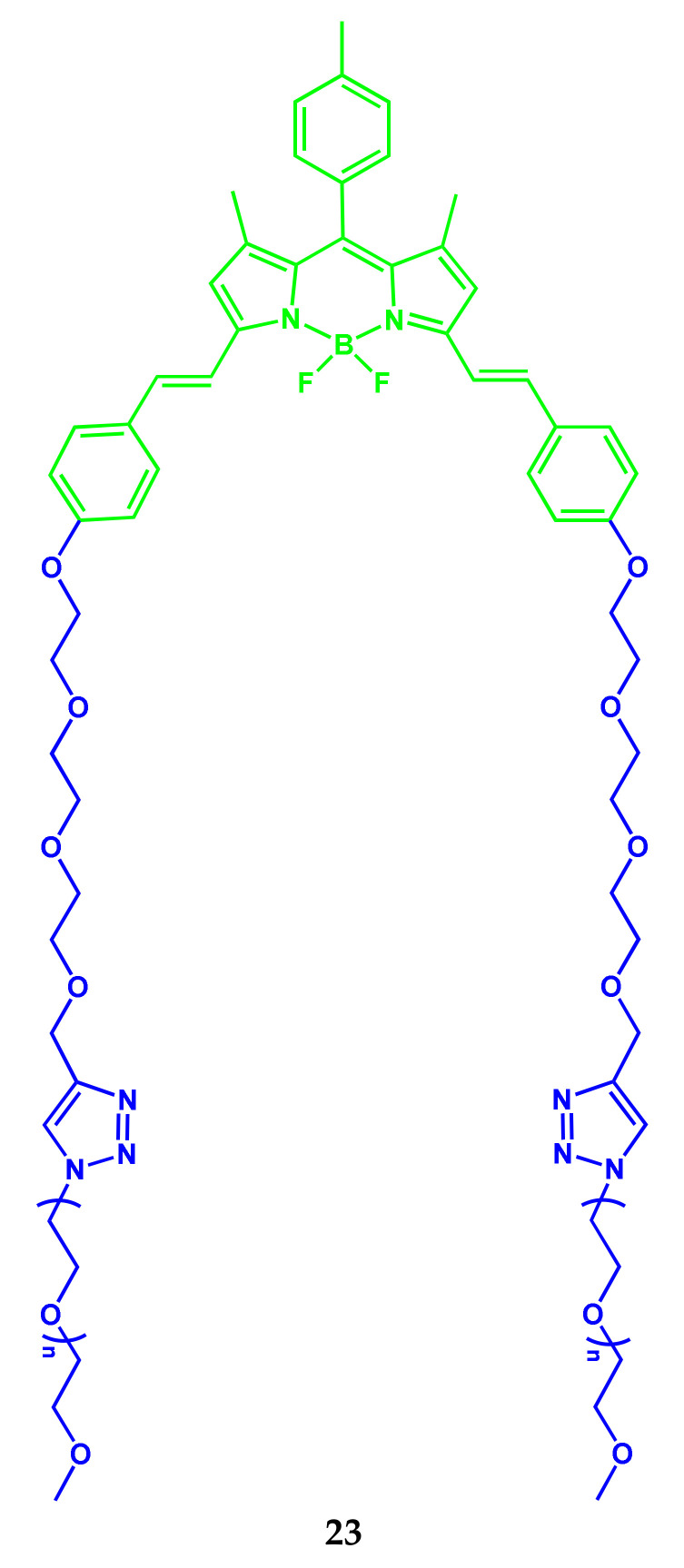
Structure of compound **23**.

Amphipathic polymer photosensitizer PEG-BODIPY (as a carrier, drug self-delivery system) **23** was synthesized (Scheme 14) for combined chemotherapy and photodynamic anticancer therapy with a real-time tracking property [19].

The conjugate **23** consists of polyethylene glycol as the hydrophilic segment and BODIPY as the hydrophobic segment. An anticancer drug such as doxorubicin can be loaded into the hydrophobic cavity of micelles, that self-assemble in an aqueous medium from two-sided amphiphilic PEGylated BODIPY polymers.

The study of the cellular uptake (MDA-MB-231 cells) of PEGylated BODIPY and Dox@PEGylated BODIPY nanoparticles showed that PEGylated BODIPY nanoparticles can be endocytosed by MDA-MB-231 cells within 24 h. The fluorescence intensity from PEGylated BODIPY increases significantly with the increasing concentration of nanoparticles. PEGylated BODIPY and Dox@PEGylated BODIPY nanoparticles were mainly concentrated in lysosomes.

**Figure 6:**
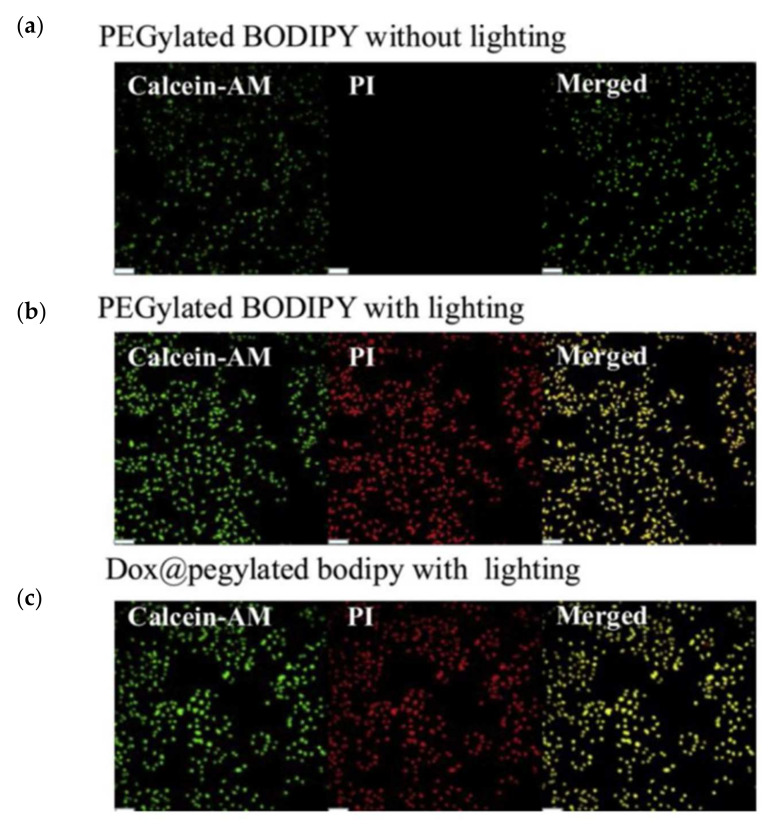
CLSM images of live/dead assay (Calcein-AM/PI) on co-stained MDAMB-231 cells after incubation with (**a**) PEGylated BODIPY without lighting, (**b**) PEGylated BODIPY with lighting, (**c**) Dox@PEGylated BODIPY with lighting. The Calcein-AM has green fluorescence and the PI has red fluorescence. Scale bar was 100 μm. Adapted from [19].

The results of studying the anticancer effects of PEGylated BODIPY and Dox@PEGylated BODIPY nanoparticles (Figure 6) demonstrated high phototoxicity and low toxicity of nanoparticles in the dark. Under illumination, the PEGylated BODIPY exhibited cytotoxicity with an IC_50_ of about 25 nM. Dox@PEGylated BODIPY exhibited even much stronger phototoxicity (IC_50_ about 10 nM). The higher IC_50_ can be explained by the combination of BODIPY photodynamic therapy and Dox chemotherapy.

The ^1^O_2_ produced by BODIPY in Dox@PEGylated BODIPY nanoparticles under light irradiation can not only cause phototoxicity to tumor cells but can also cause disassembly of Dox@PEGylated BODIPY nanoparticles, which leads to the release of Dox to reach the tumor nucleus for chemotherapy.

Thus, harvested micelles can also work as nanocarriers for chemotherapy drugs such as Dox to achieve combined photodynamic and chemotherapeutic anticancer treatments.

**Scheme 15:**
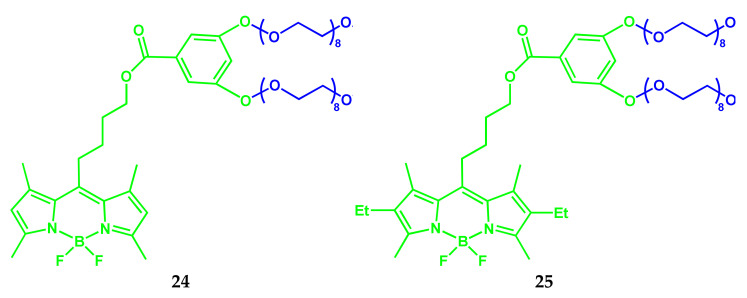
Structures of compounds **24** and **25**.

A series of water-soluble BODIPY dyes **24** and **25** modified by PEG were synthesized and investigated in various solutions (Scheme 15) [20].

**Figure 7:**
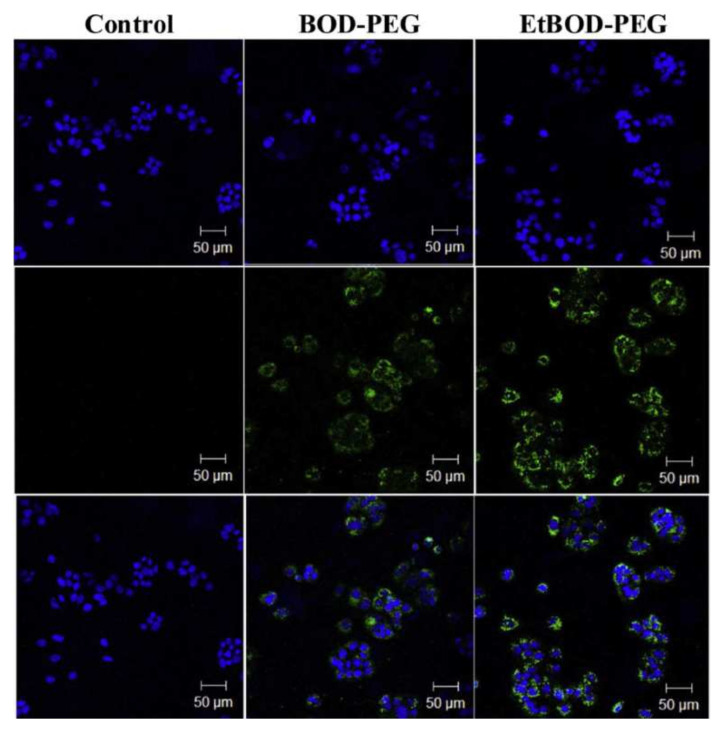
CLSM images of MCF-7 cells with BODIPY-PEG **24** or Et-BODIPY-PEG **25** at various concentrations, with the nucleus labeled with 4′,6-diamidino-2-phenylindole (DAPI). Top: DAPI images; middle: BODIPY-PEG/Et-BODIPY-PEG dye images; bottom: merged images of both (10.0 μM). Adapted from [20].

The introduction of bulky bi-branched PEG chains into the meso-position of the BODIPY nucleus led to an increase in the solubility and fluorescence quantum yields in water, and also reduced the tendency of luminophors to aggregation. At the same time, PEG has no significant effect on the photophysical properties of BODIPY in the conjugate. The values of the absolute fluorescence quantum yields of PEG-BODIPY conjugates **24** and **25** in organic solvents are close and amounted to 0.624–0.681. For conjugate **24** with β-unsubstituted BODIPY, the Φ_F_ in water is 0.512, which is two times higher, than the Φ_F_ of conjugate **25** with β,β′-diethyl-substituted BODIPY (Φ_F_ 0.254). Moreover, with an increase in the concentration of conjugate **25**, the values of its fluorescence quantum yield in water noticeably decrease, which is associated with aggregation in an aqueous solution. After 24 h incubation with fluorescent dyes, no significant decrease in cell viability was observed (Figure 7).

The BODIPY conjugates **24** and **25** showed no toxicity to MCF-7 cells at low concentrations for 24 h. The dyes are permeable to MCF-7 cells and are concentrated in the area of the cellular cytoplasm. The results obtained using confocal microscopy confirm the prospects of using conjugates in fluorescent bioimaging.

**Scheme 16:**
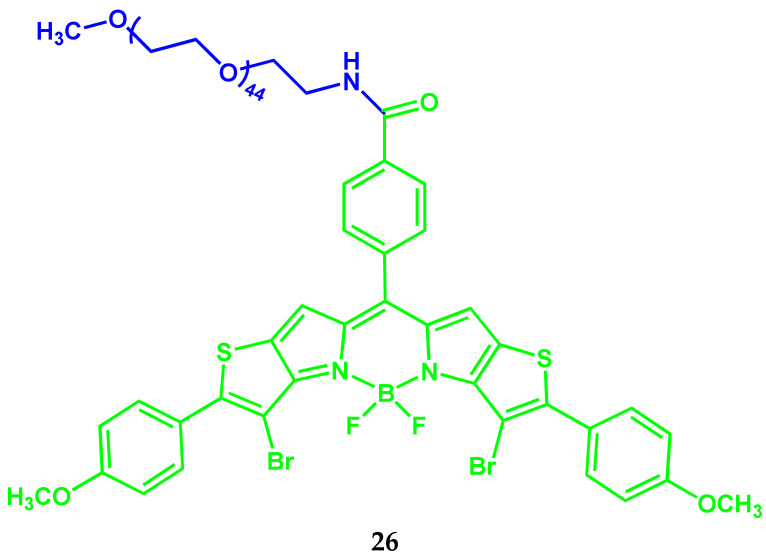
Structure of compound **26**.

By conjugating the modified BODIPY with hydrophilic PEG, the amphipathic polymer PEG-BODIPY **26** was synthesized as an effective PDT agent with “favorable” phototoxicity against HepG2 and 4T1 cell lines (Scheme 16) [21].

Amphiphilic macromolecules of **26** can self-assemble into micelles with a suitable size, not only for long-term circulation in the body but also for in vivo accumulation in the tumor area. The **26** works as a photosensitizer and fluorescent probe, demonstrating excellent treatment and imaging capabilities both in vitro and in vivo.

### 2.5. BODIPY Conjugate with Chlorambucil

Chlorambucil is an antitumor bifunctional alkylating agent, a derivative of aromatic nitrogen mustard. Alkylation of cells occurs through the formation of a highly reactive ethyleneimonium radical between two strands of the DNA helix, with subsequent interference with replication. In therapeutic doses, it inhibits the synthesis of lymphocytes, while exerting a less pronounced effect on neutrophils, platelets, and erythrocytes.

**Scheme 17:**
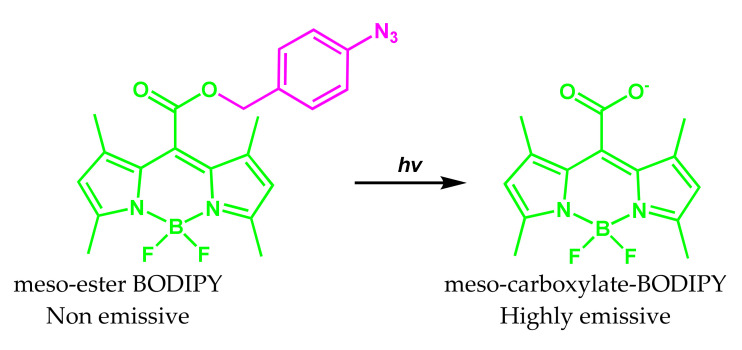
Conversion of meso-ester-BODIPY into meso-carboxylate-BODIPY.

Photoactivated fluorophore [22], the action of which is based on the conversion of the non-fluorescent meso-ester-BODIPY into the fluorescent meso-carboxylate-BODIPY with a noticeable difference in fluorescence between the cellular and extracellular state, was developed (Scheme 17).

The fluorescence quantum yield of meso-carboxylate-BODIPY becomes 70 times higher than the quantum yield of the meso-ether-BODIPY.

**Scheme 18:**
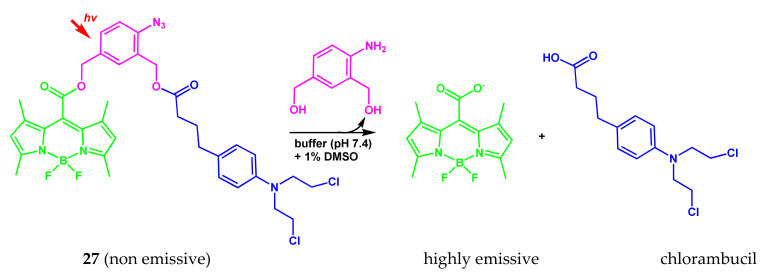
Structure of compound **27** and release process of drug molecule and fluorescent dye upon light activation.

The authors of [22] propose the BODIPY conjugate with the anticancer drug chlorambucil **27** as a universal photoactivated platform for bioactive molecules. Upon light activation of **27** in water (Scheme 18), both the bioactive drug molecule and the fluorescent dye are released, which leads to an increase in luminescence by about 1250 times, including in living cells.

The **27** proved to be chemically stable under assay conditions and resistant to potential chemical attack, ensuring, that the release of meso-carboxylate-BODIPY fluorophore is only achieved by photochemical activation of the arylazide group.

### 2.6. BODIPY Conjugates with Isoxazole

Isoxazole forms the basis of a number of drugs. Isoxazole is part of many natural biologically active compounds. Its derivatives are used to treat diseases of the central nervous system, as antitumor agents, analgesics, anesthetics, muscle relaxants, antibiotics.

**Scheme 19:**
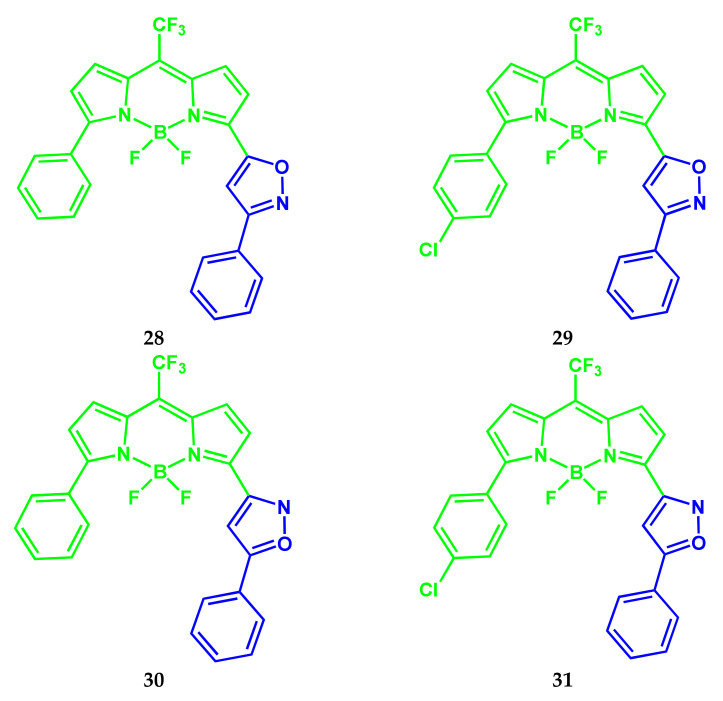
Structures of compounds **28**–**31**.

Two approaches were developed for the synthesis of meso-CF_3_-substituted BODIPY dyes **28**–**31** containing an isoxazole substituent at the α-position of the BODIPY nucleus (Scheme 19) [23].

The new BODIPY dyes **28**–**31** are characterized by promising optical characteristics: absorption and fluorescence in the regions of 584–617 nm and 611–646 nm, respectively, and high (from 0.69 to 0.94) fluorescence quantum yield. The conjugation of the BOIDPY core with biologically and pharmaceutically important isoxazole rings opens up new promising opportunities for drug design and application in photodynamic therapy.

### 2.7. BODIPY Conjugate with Capsaicin 

Capsaicin inhibits the growth of several types of cancer, including adenocarcinoma (the most common type of lung cancer), melanoma, and cholangiosarcoma (a type of liver cancer), and prevents metastasis.

**Scheme 20:**
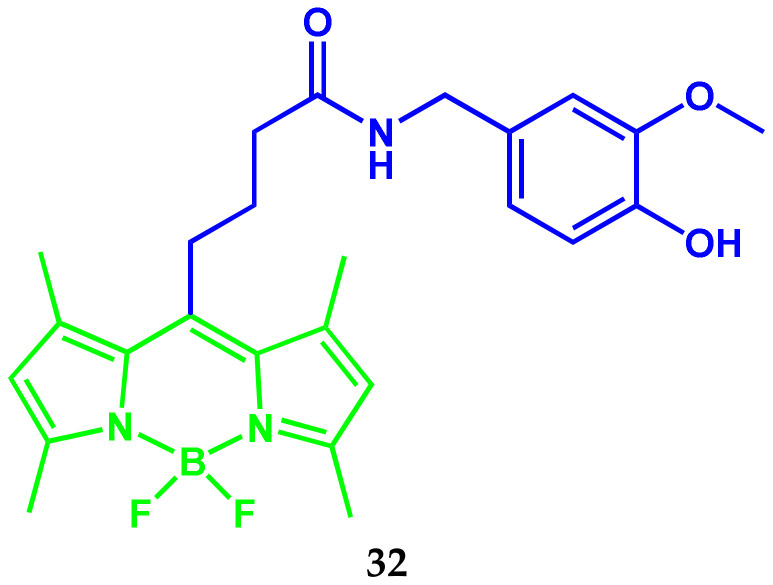
Structure of compound **32**.

Angel Sampedro and colleagues proposed a strategy to improve the antitumor effect of capsaicin in prostate cancer by preparing a covalent conjugate with BODIPY **32** (Scheme 20) [24]. The improvement in the antitumor effect is based on enhanced permeability and retention by increasing the aggregation capacity of capsaicin in aqueous media. The conjugate **32** self-assembles in aqueous solutions to form weakly fluorescent globular nanoclusters. Upon cellular uptake, nanoclusters are disassembly and become high-emissive.

Conjugate **32** in monomeric form does not have a significant antitumor effect in vivo in prostate cancer, while aggregation into nanoclusters sharply increases the accumulation of tumors. It was found that effective doses can be reduced by two orders of magnitude (18 mg/kg) compared to unmodified capsaicin while maintaining comparable antitumor activity against prostate cancer cell line PC-3.

### 2.8. BODIPY Conjugates with Mertansine

Mertansin is a thiol-containing maytansinoid that is therapeutically coupled to a monoclonal antibody by reacting a thiol group with a linker structure to create an antibody–drug conjugate. The antibody–drug conjugate exhibits potential antitumor activity.

**Scheme 21:**
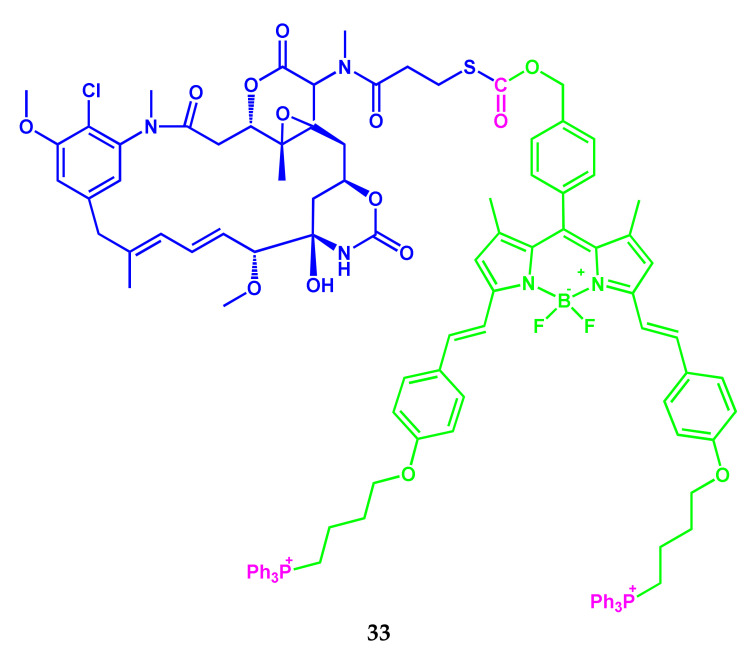
Structure of compound **33**.

Recently, Rajeshwari Tiwari and colleagues reported a prodrug conjugate **33** (Scheme 21). The conjugate consists of the antitumor drug mertansine and the NIR-active BODIPY-luminophor, covalently linked by a light-cleavable thioether bond [25].

**Figure 8:**
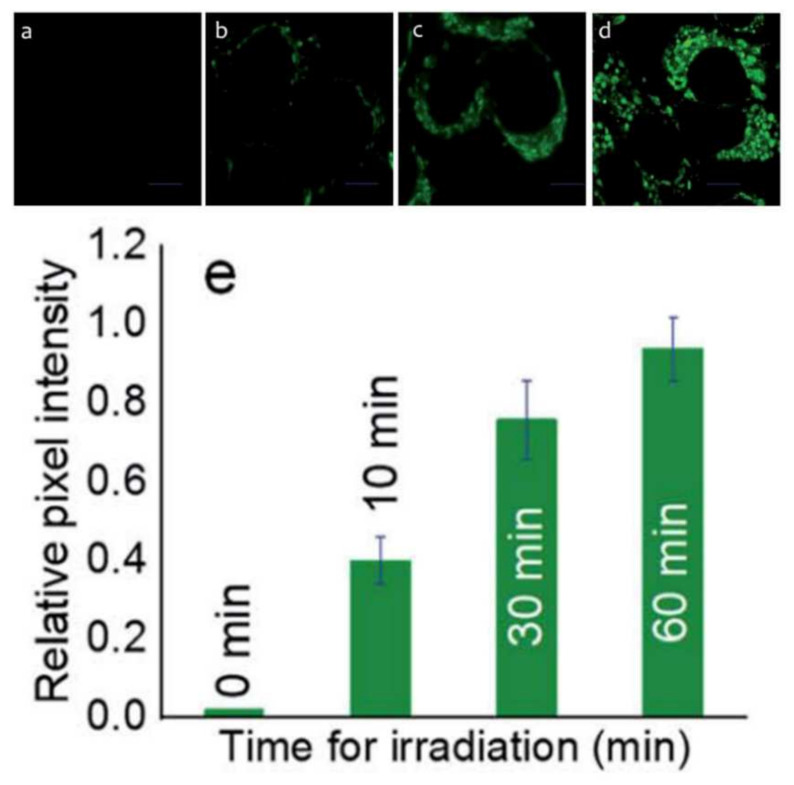
Cellular fluorescence images of prodrug **33** treated MCF-7 cells. The cells were incubated with **33** (1 mM) for 2 h and then irradiated with a blue LED for: (**a**) 0 min, (**b**) 10 min, (**c**) 30 min, and (**d**) 60 min. (**e**) Relative pixel intensity (*n* = 3) from the images. The pixel intensity from the images. The pixel of (**d**) is defined as 1.0. Scale bar 10 μm. λ_ex_ 650 nm; λ_Mon_ window of 530–710 nm. Adapted from [25].

Under light irradiation by visible light, the prodrug **33** undergoes a photodegradation reaction to simultaneously release therapeutic components of mertansine and CO for chemotherapy, together with a BODIPY marker for biovisualization of intracellular luminescence in MCF-7 cell organelles (Figure 8).

The results of the authors’ studies [25] confirm the possibility of developing a prodrug for combination therapy with visual control for cancer treatment. The molecular design of such a prodrug provides several functions at once, including targeting, imaging, release, and treatment, which undoubtedly play an important role in biomedicine.

### 2.9. BODIPY Conjugates with Terpenes

**Scheme 22:**
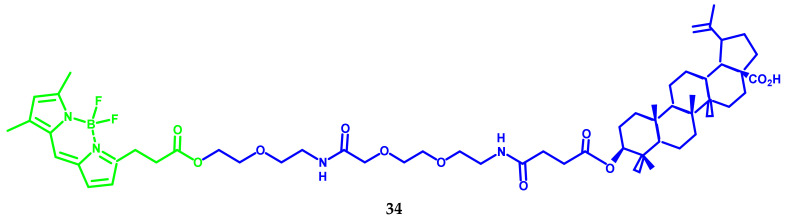
Structure of compound **34**.

The design of new natural biologically active substances and the study of the mechanism of their action are some of the most important tasks of modern biochemistry and biomedicine. Of particular interest is the search for new compounds with high antitumor and antithrombotic activity, selectivity of action on the pathogenic microorganisms, etc. The natural monoterpenes, their synthetic analogs, and derivative conjugates with other bioactive compounds are of particular interest [26,27,28,29]. Presently, the antifungal activity of *α*- and *β*-pinenes [30] is well studied, and there is information about the antifungal effect of essential oils, including verbenols and myrtenol [31,32,33]. It has been shown that thioterepenoids also have anti-helicobacter, antiaggregatory, and anticoagulant effects. In addition, this class of compounds is characterized by low toxicity, mutagenic, and genotoxic effects [34,35,36,37]. In 2018 and 2019, the authors of [38,39] presented a first paper dealing with the fluorescence labeling of terpenes by BODIPYs. It was shown that the cytotoxicity of the triterpene BODIPY conjugates strongly depended on the spacer between the triterpenoid core and the BODIPY FL unit. Among the studied compounds, only the 3-O-acetyl-betulinic acid-derived BODIPY FL conjugate **34** (Scheme 22) holding an ethylendiamine spacer was cytotoxic for human breast adenocarcinoma cells MCF7 but not cytotoxic for all other cell lines.

**Scheme 23:**
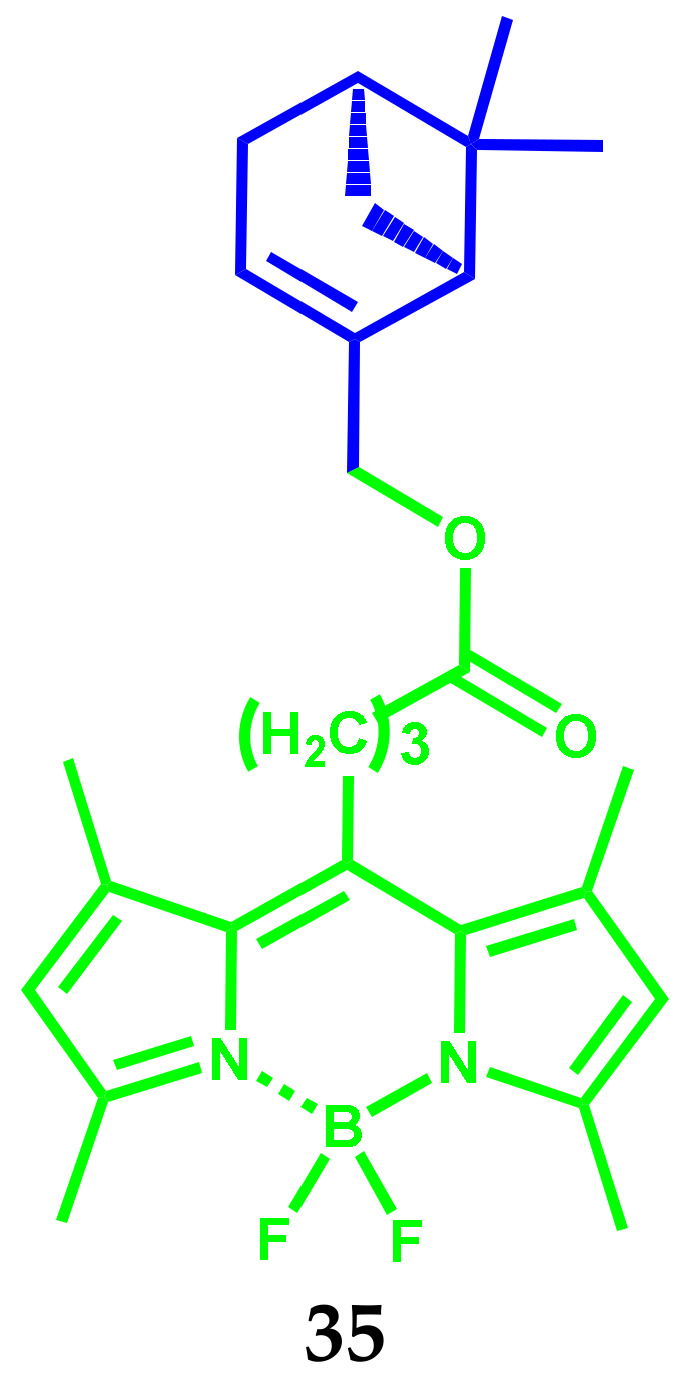
Structure of compound **35**.

Recently, the authors [40] presented the results of spectral and biological studies of the BODIPY phosphor with myrtenol **35** (Scheme 23).

**Figure 9:**
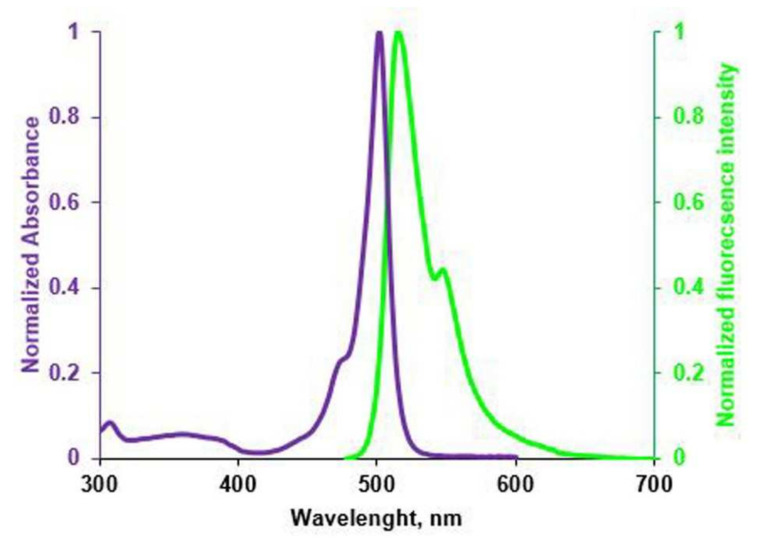
The normalized absorption (purple) and emission spectra (green) of BODIPYmyrt in 1-octanol.

Studies have shown that the conjugate efficiently absorbs (*ɛ* ~10^5^ mol∙L^−1^) and fluoresces (from ~75 to ~100%) regardless of the medium properties in the region at 500–518 nm (Figure 9).

**Figure 10:**
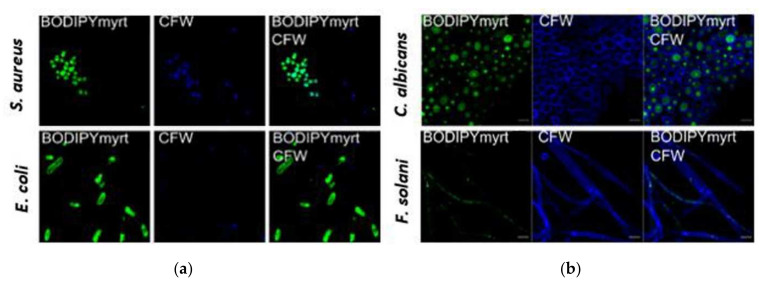
CLSM of bacterial cells (**a**) and fungal cells (**b**) stained by BODIPYmyrt and calcofluor-white (CFW). Scale bar is 10 μm. Adapted from [40].

The conjugate exhibits a tendency to rapidly penetrate into bacterial, mammalian, and fungal cells and bind with membranes. Strong fluorescence was detected only in BODIPY-treated *S. aureus* cells, assuming no accumulation of conjugate in the membranes (Figure 10). By contrast, in *E. coli*, the pronounced green fluorescence was observed on the cell edge suggesting that the combination of myrtenol with BODIPY led to no penetration into the cell. Green fluorescence was also recorded in the cells of *C. albicans* fungi, while BODIPY was associated mainly with the cell nucleus, and in the threads of *F. solani* BODIPY, the conjugate was concentrated in the form of rare spots (Figure 10).

The authors believe that the covalent binding of myrtenol to the BODIPY fluorophore promotes selective staining of specific cell organelles, which can be used to determine the cellular localization of a compound of interest. Meanwhile, the cellular targets of the conjugate remain to be elucidated. The first results obtained allow us to expect that research will provide a fruitful basis for the development of new effective terpene conjugates with improved biologically active properties.

## 3. BODIPY Conjugates for Bioimaging

Along with the multifunctional conjugates of BODIPY-fluorophore with anticancer drug compounds described in Section 2, the conjugates that act primarily as fluorescent markers for bioimaging are being actively developed. Such compounds allow bioimaging for the transport of molecules to target cells, their localization in organelles, redox, and other metabolic processes. For example, protein oligomers, which are formed as a result of the aggregation of protein molecules under physiological stress, are neurotoxic and responsible for a number of neurological diseases. Therefore, the detection of protein oligomers is essential for timely intervention in concomitant diseases. Although several probes for the detection of insoluble matured protein fibrils were developed, the NIR fluorescent probes for the study of protein oligomers are very rare. Next, we will consider BODIPY conjugates with various biomolecules, including vitamins, glucose, steroids, lipids, which function in living organisms as fluorescent markers for bioimaging, transport of a compound to target cells, etc.

### 3.1. BODIPY-Conjugates with Vitamins

Given the critical role of vitamins in living systems, non-invasive imaging techniques are highly desirable to monitor the redox and transport properties of vitamins in real time and in situ. One of the preferred methods of monitoring transport and metabolism of vitamin is based on its conjugation with a fluorophore, which allows the using fluorescence microscopy. 

**Scheme 24:**
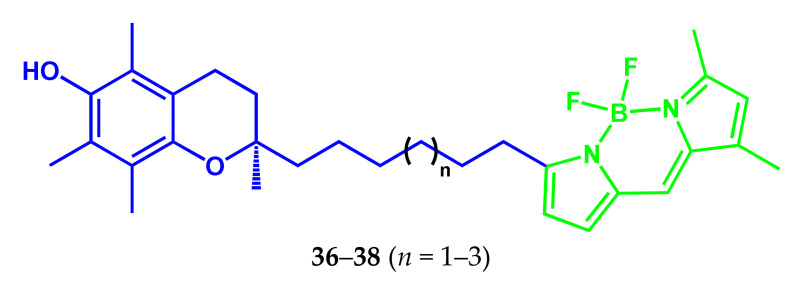
Structures of compounds **36**–**38**.

In 2010, conjugates of three fluorescent analogs of R-tocopherol with the BODIPY fluorophore (C_6_-, C_7_- и C_8_-BODIPY-R-Toc) **36**–**38** were synthesized (Scheme 24) as probes in the study of the localization and intracellular transport of vitamin E [41].

For **36**–**38** to be of any “utility”, they must demonstrate an affinity to the tocopherol (R-TTP). The dissociation constants were estimated from the results of fluorescence titration of R-TTP with **36**–**38**. The C_8_-BODIPYR-Toc **38** (λ_ex_ = 507 nm, λ_em_ = 511 nm, ε = 83,000 M^−1^ cm^−1^) has the best affinity to the R-TTP (K_d_ = 94 nM). The K_d_ values for reactions with two other conjugates (C_7_-BODIPYR-Toc and C_6_-BODIPYR-Toc) are significantly higher and amounted to 130 and 232 nM, respectively. The dissociation constant for α-tocopherol transfer protein (α-TTP), the natural ligand for R-TTP, is 25 nM [42].

**Scheme 25:**
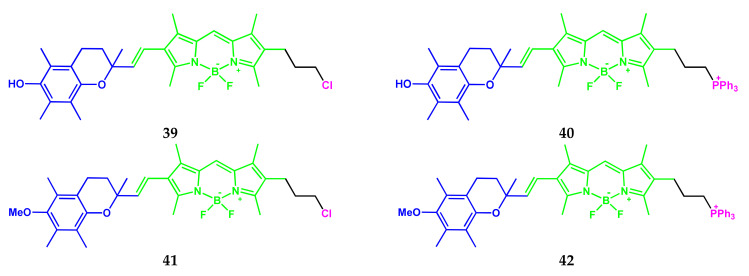
Structures of compounds **39**–**42**.

Another example of BODIPY-tocopherol conjugates **39**–**42** (Scheme 25) as a mitochondria-targeting fluorogenic antioxidant was demonstrated in [43]. The methodology for the synthesis and characteristics of lipophilic fluorogenic antioxidants, analogs of α-tocopherol–probes **39**–**42** for live-cell imaging were described. Conjugates consist of an α-tocopherol-like chromanol fragment (receptor), a BODIPY fluorophore (reporter), and an element targeting mitochondria (triphenylphosphonium cation).

In terms of structure and reactivity, the probes **39**–**42** are analogs of the most powerful natural acceptor of free radicals of α-tocopherol and are designed to targeting on the internal mitochondrial membrane.

The maxima of the absorption and fluorescence spectral bands of the conjugates **39**–**42** are located in the regions of 536–537 and 572–585 nm, respectively. Hydroxysubstituted compounds **39** and **40** showed a fluorescence quantum yield 10 times lower, than for methoxysubstituted compounds **41** and **42**.

Conjugates are sensitive to the presence of lipid peroxyl radicals, which are effective chain carriers in the process of lipid chain autoxidation. Moreover, conjugates **39**–**42** and α-tocopherol have the same reaction rate constant with peroxyl radicals. Kinetic studies clearly indicate the similar antioxidant activity of α-tocopherol and **39**–**42** and highlight that **39**–**42** were embedded in the membrane in much the same way as α-tocopherol. The results on the localization of dye-conjugates in NIH 3T3 cells showed that the lipophilic fluorogenic antioxidant **40** and its fluorescent analog **42** accumulate mainly in the internal mitochondrial membrane due to the lipophilic cationic segment (phosphonium label) and common lipophilic part.

The results of studies on living cells showed, that **39**–**42** allows real-time monitoring of the generation of lipid peroxyl radicals and the depletion of antioxidant properties in the internal mitochondrial membrane. The authors of [43] note the potential of the fluorogenic antioxidant **39**–**42** to investigate the relationship between antioxidant load, the onset of lipid chain autooxidation, mitochondrial physiology, and mitochondrial dysfunction.

**Scheme 26:**
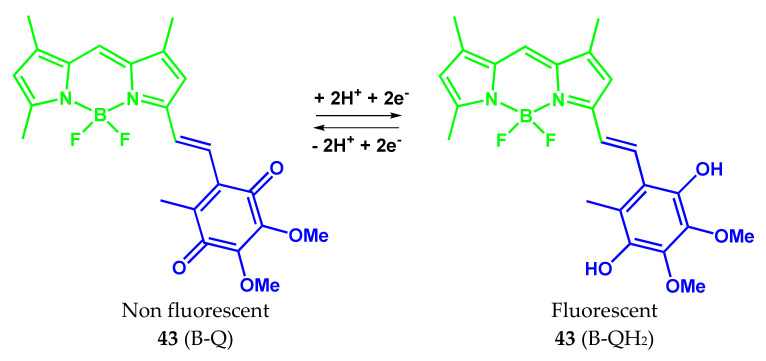
The oxidized and reduced forms of compound **43**.

The design, preparation, and characterization of a two-segment fluorogenic analog of ubiquinone **43** (Scheme 26) for monitoring red-ox reactions in biological systems were reported in the [44]. The conjugate **43** consists of the redox-active 2,3-dimethoxy-1,4-benzoquinone (receptor), covalently linked to the lipophilic BODIPY fluorophore (reporter). As a result, the PeT-sensitive probe does not fluoresce in oxidized form but becomes fluorescent in reduced form, when the quinone moiety is converted to dihydroquinone.

The electronic absorption and fluorescence spectra of conjugate **43** in the oxidized and reduced forms are in the range 572–595 nm, depending on the medium nature. The band positions of the conjugate **43** containing the monostyryl moiety are markedly red-shifted due to the extended conjugation contour in styryl-BODIPY, compared, for example, with the alkyl-substituted analogs.

The reduced form of the conjugate **43** exhibits intense fluorescence in non-polar solvents (toluene and dichloromethane) with a quantum yield of 0.66 and 0.55, respectively. In polar acetonitrile, the quantum yield decreases to 0.01. Presumably, in polar solvents, the PeT process from the HOMO of the dihydroquinone fragment to the photoexcited BODIPY segment easily occurs. The developed probe **43** demonstrates chemical and electrochemical reversibility, which is critical for monitoring redox reactions. The antioxidant behavior of the reduced form of conjugate **43** and its synergistic activity with chromanol (the active segment of vitamin E) in trapping peroxyl radicals were studied. The results of research [44] have shown that the conjugate **43** can serve as a candidate for the study and understanding of complex redox reactions in non-polar media and also serve as a model for the development of second-generation probes with the necessary sensitivity for monitoring electron transfer processes in mitochondria.

**Scheme 27:**
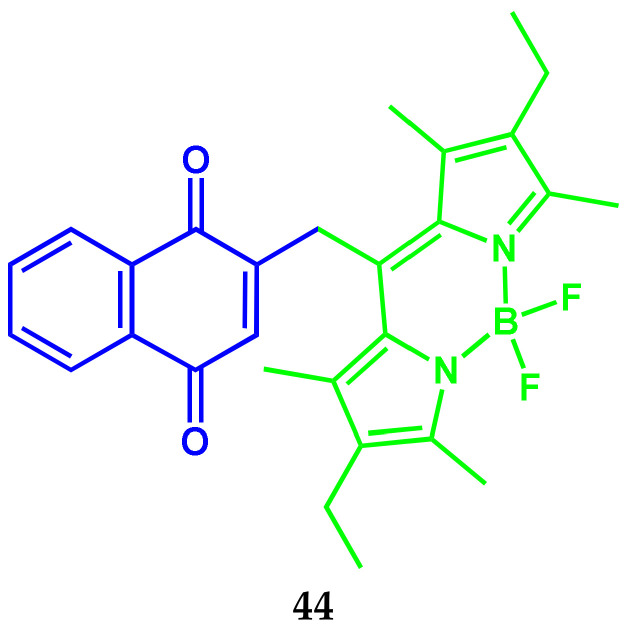
Structure of compound **44**.

In contrast to the fluorogenic BODIPY conjugate with the ubiquinone [44], the conjugate **44** presented in [45] significantly exceeds it by sensitivity (Scheme 27). The two-component fluorogenic conjugate **44** consists of vitamin K3 (menadione or 2-methyl-1,4-naphtoquinone, as quinone redox center), binding to the meso-position of BODIPY fluorophore (lipophilic reporter segment) through the methylene linker.

The absorption and fluorescence spectra of conjugate contain bands with maxima at 522–532 nm and 536–546 nm, respectively. The extinction coefficients of conjugate **44** are in the range of 21,000–85,000 M^−1^ cm^−1^. The probe action is based on intramolecular PeT from the BODIPY fragment to the naphthoxinone receptor to ensure that fluorescence is turned off. The fluorescence quantum yield of conjugate in the oxidizes form is 0.0006 in toluene. In the restoration of the menadione segment to menadiol, a high-emission dihydroquinone form of probe (conjugate **44**-H_2_) with a fluorescence quantum yield of about 0.54 in toluene is formed. Thus, **44** demonstrates high sensitivity, superior to a 1000-fold increase in fluorescence in non-polar systems, which makes it a suitable probe for enzymatic studies. The biocompatibility of conjugate **44** was demonstrated in reactions with D,T-diaphorase and by introducing it into a lipid membrane. The enzymatic studies showed that the probe **44** is compatible with biological systems and can serve as an electronic shuttle, operating in tandem with proteins. Conjugate is suitable for monitoring the transfer and transport of electrons in model systems and biological structures. 

**Scheme 28:**
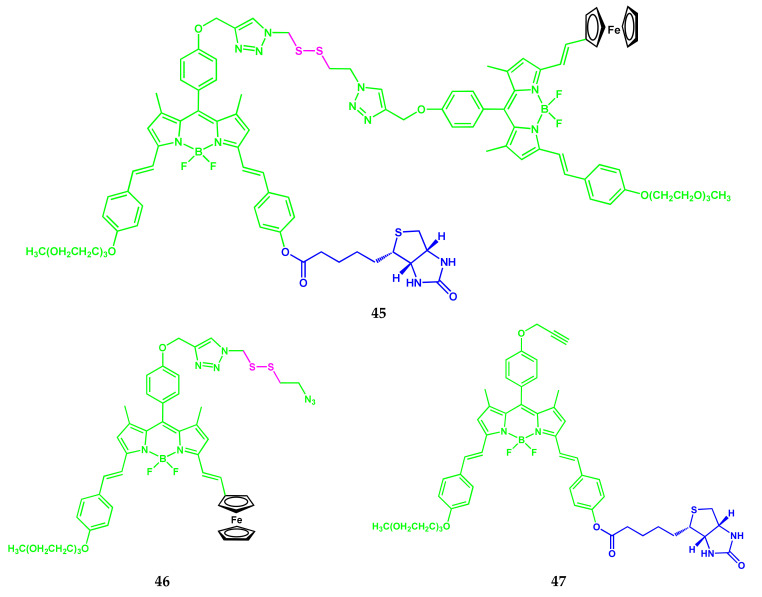
Structures of compounds **45**–**47**.

The fluorescent probe in the form of FRET-conjugate **45** based on ferrocenyl-BODIPY **46** containing a disulfide group and biotin-distyryl-substituted BODIPY **47** was synthesized (Scheme 28) [46]. Biotin was used as a potential guide ligand for cancer cells.

The three bands with maxima at 638 nm, 582 nm (vibronic band), and 362 nm are presented in the electronic absorption spectra of **47** in DMF. The **47** shows an intense fluorescence at 648 nm. Conjugate **45** showed a typical absorption spectrum with the maxima of the bands at 638 nm, 583 nm, and 362 nm, characteristic for distyryl-substituted BODIPY, and its fluorescence is weaker (by 6 times) than that of **47**. Fluorescence quenching of **45** was also found in PBS (with 0.5% Tween 80). Differences in fluorescence of conjugates **47** and **45** can be observed with the naked eye.

The logarithms of the extinction coefficient of conjugates **45** and **47** are in the range from 4.54 to 5.04. In the presence of 2 mM glutathione, imitating the intracellular medium, the absorption spectrum of **45** in PBS was not changed significantly, in contrast to the fluorescence spectra. Fluorescence intensity was gradually increased over 10 h. The reduction of fluorescence can be explained by the splitting of the disulfide linker by glutathione and the separation of the distyryl BODIPY fluorophore and the ferrocenyl quencher. Conjugate **45** demonstrates the preferred cellular uptake by cells A549, compared with the CNO-K1 cells.

**Scheme 29:**
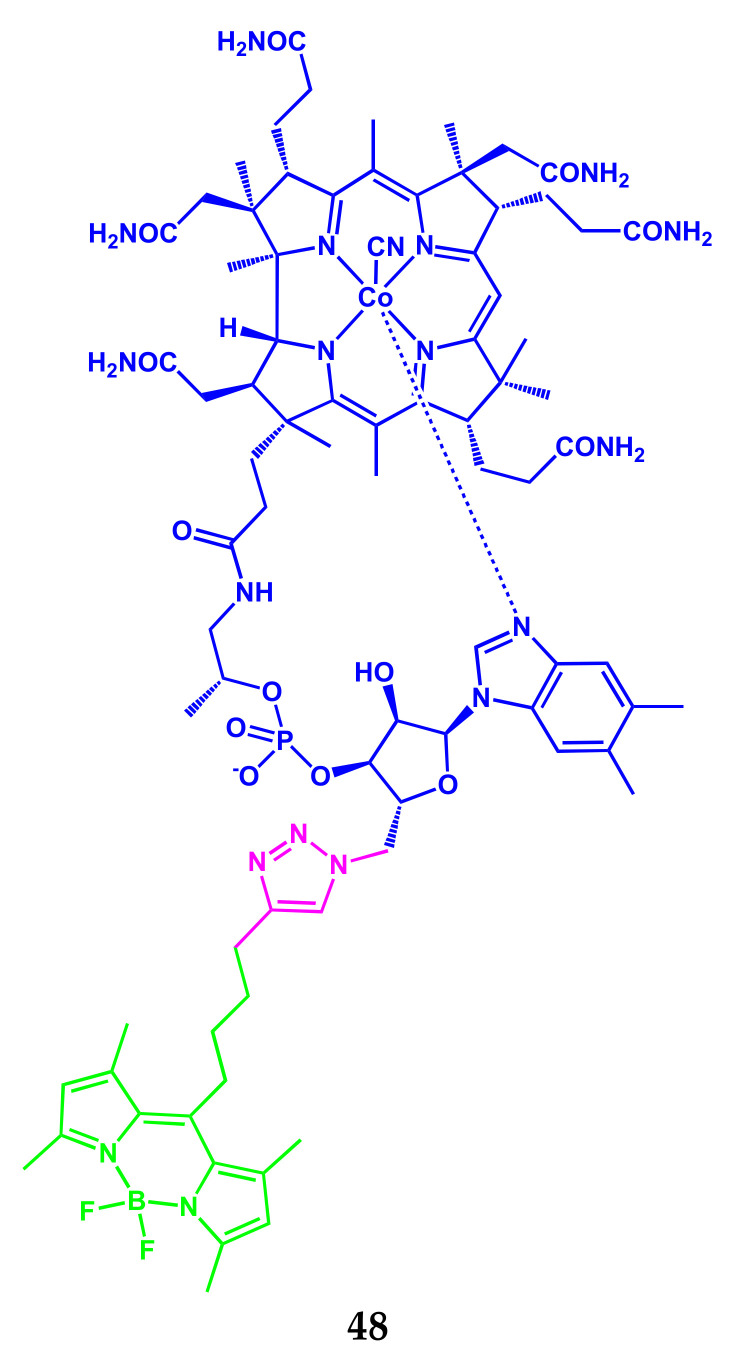
Structure of compound **48**.

The development and characteristics of the stable fluorescent conjugate **48** of vitamin B_12_ (cobalamin) with the BODIPY fluorophore (Scheme 29) were demonstrated in [47].

UV–Vis spectrum of BODIPY conjugate with vitamin B_12_ is a simple overlap of the absorption spectra of alkyl-substituted BODIPY and vitamin B_12_. The absorption spectrum of the conjugate **48** contains the characteristic bands of the corrin ring of the vitamin B12 at 360 nm (ε = 56,000 M^−1^ cm^−1^) and 552 nm (ε = 19,550 M^−1^ cm^−1^), as well as the transition *S*_o_ → *S*_1_ from BODIPY at 493 nm (ε = 68,350 M^−1^ cm^−1^). A solution of vitamin B_12_ labeled by BODIPY has a fluorescence maximum at 508 nm (with λ_ex_ 493 nm) with a fluorescence quantum yield of 0.1 in water.

**Figure 11:**
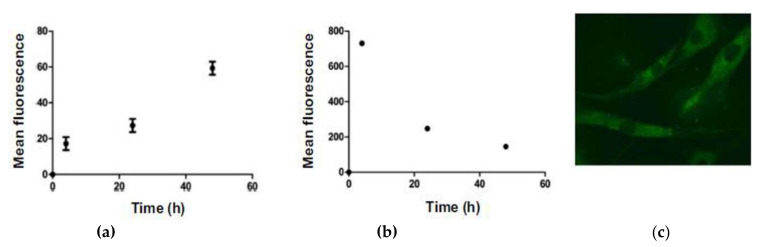
Flow cytometric analysis of human fibroblasts cells incubated for 4, 24, or 48 h with 20 μM BODIPY-labeled vitamin B_12_ (**a**) or free BODIPY (**b**). Fluorescence microscopy images of human fibroblasts cells incubated 24 h with free BODIPY (**c**). Adapted from [47].

Flow cytometry studies showed, that vitamin B_12_ in conjugate with BODIPY was absorbed by cells for 48 h, while the non-conjugated BODIPY did not accumulate in cells (Figure 11). Vitamin B_12_ binds to transcobalamin protein, which is necessary for the transport of vitamin B_12_ to target tissues.

The results of confocal microscopy showed that vitamin B_12_ labeled by BODIPY demonstrates cellular internalization and accumulation. In vitro studies on wild-type human fibroblasts showed that vitamin B_12_ labeled by BODIPY can be absorbed in the same way as non-labeled vitamin B_12_. Fluorescent conjugate **48** can be potentially suitable for use as an easily tracked probe to study the transport of vitamin B_12_ to cells and the intracellular behavior of vitamins in the context of disorders associated with the anomalous use of vitamins in cells.

**Scheme 30:**
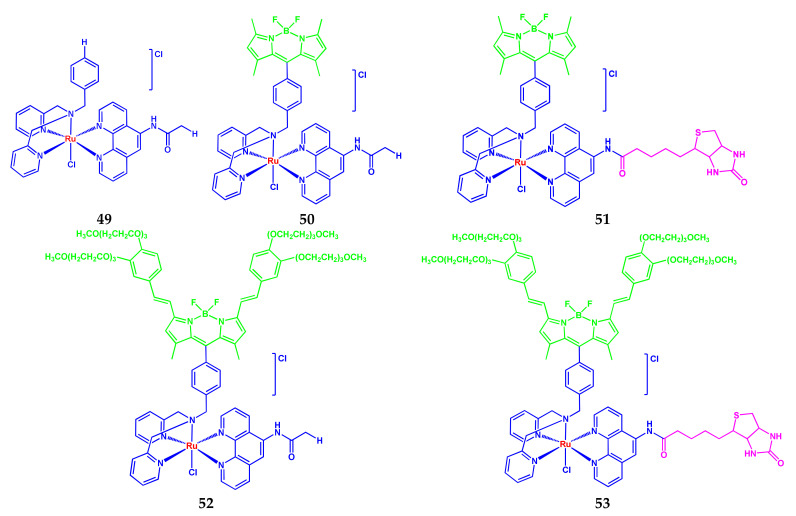
Structures of compounds **49**–**53**.

Another interesting example of the development and study of multicomponent BODIPY conjugates **49**–**53** (Scheme 30) was described by Subhadeep Paul with colleagues in a published article 2020 [48]. The authors of [48] studied the cell localization and photocitotoxicity of a new class of ruthenium(II) complexes, formed by NNN-dipicolylamin bases, containing a modified nucleus of photoactive BODIPY and NN-phenanthroline derivatives with biotin fragment.

Adding a biotin fragment as vitamin B_7_ to the structure improved the photocitotoxic effect in A549 cells and gave greater selectivity against cancer cells compared to normal cells.

Absorption spectra of conjugates **49**–**53** in DMSO/DPBS buffer are noticeably different. The spectrum of complex **49** (in the absence of BODIPY) was characterized by a broad band with a relatively low absorption intensity (11,000 M^−1^ cm^−1^) and maxima of about 464 and 350 nm due to MLCT transitions (transfer of charge from metal to ligand). Complexes **50** and **51** containing the tetramethyl-substituted BODIPY showed the intensive absorption peak at 500 nm (ε 58,000–61,000 M^−1^ cm^−1^), attributed to the π–π* transition of BODIPY. Complexes **52** and **53** containing a distyryl-substituted BODIPY fragment demonstrated intensive absorption in the phototherapeutic window at 654 nm with high values of the extinction coefficient (80,000–88,000 M^−1^ cm^−1^). The fluorescence spectra of conjugates in DMSO/DPBS buffer contain one band with a different position and intensity of the band maxima. Conjugate **49** shows a weak fluorescence. Complexes **50** and **51** have emission bands with a maximum at 508 nm with a small Stokes shift. The values of the relative fluorescence quantum yield in DMSO for these complexes were 0.09 and 0.08, respectively. Complexes **52** and **53** with fluorescence maxima at 671 nm (λ_ex_ = 630 nm) have the values of Φ_F_ 0.07 and 0.06. Conjugates in 10% DMSO/DPBS are resistant in the dark under observations up to 48 h. With light (400–700 nm) irradiation, complexes **50** and **51** showed a slight decrease in the absorption, recorded at a maximum at 500 nm. A noticeable decrease in the absorption intensity was observed for **52** and **53**, due to the higher efficiency of singlet oxygen generation by these complexes. The ΦΔ values for conjugates were 0.18 (**50**), 0.22 (**51**), 0.64 (**52**) and 0.65 (**53**). The binding constants *K*_b_ of conjugates with ct-DNA in a 10% DMF-Tris-HCl buffer (pH 7.2) are from 0.82 × 10^5^ to 2.7 × 10^5^ M^−1^. Complexes **52** and **53** containing distyryl-BODIPY showed higher binding constants, compared to **50** and **51**. In turn, higher binding constants for biotin-containing conjugates **51** and **53**, compared to **50** and **52**, can be associated with additional DNA interactions with side biotynamide groups.

Using the example of complex **53**, the ability to split DNA in the presence of light using supercoiled (SC) pUC19 DNA (30 μm, 0.2 μg) in Tris-HCl/NaCl (50 mm, pH 7.2) buffer was studied. The complex showed a significant ~65% splitting of SC DNA in the presence of 33 μm of **53** and irradiation with monochromatic light for 15 min, using a diode laser with a wavelength of 660 nm (100 MW). At the same time, the complex did not show the visible splitting of DNA in the dark (<5%), which indicates its minimal toxicity in the dark. 

Analysis of photocitotoxicity of complexes using A549 and HPL1D cells showed that complexes are non-toxic in the dark (IC_50_ > 100 μM). When irradiated for 1 h, complexes **50** and **51** showed moderate photoinduced cytotoxicity with the values of IC_50_ ~2.0 and 0.98 μM in the A549 cells in the visible light of 400–700 nm. Photocytotoxicity of complexes **50** and **51** towards HPL1D cells decreased by about four times under the same conditions of photoirradiation. For complex **52** after light activation for 15 min the IC_50_ value was 0.04 mM. For biotin-containing conjugate **53** IC_50_ value is 0.02 μM. Complexes **52** and **53** are relatively less toxic for HPL1D cells, giving IC_50_ are 0.13 and 0.09 μM.

**Figure 12:**
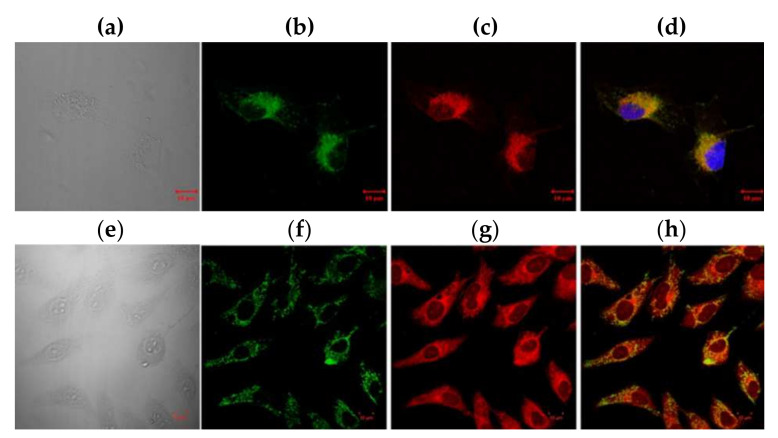
Confocal microscopic images of A549 cells after 4 h of incubation with **51** and **53**. The top panels: (**a**) bright field, (**b**) fluorescence of **51**, (**c**) fluorescence of Mitotracker deep red (MTR), (**d**) merged image of **51**, Mitotracker deep red and Hoechst. The bottom panels: (**e**) bright field, (**f**) fluorescence of Mitotracker green (MTG), (**g**) fluorescence of **53**, and (**h**) merged image of **53** and Mitotracker green. The overlap coefficient of **51** with Mitotracker red is ∼0.65, and that for **53** and Mitotracker green is ∼0.6. Scale bar represents 10 μm. Adapted from [48].

The cellular uptake of biotin-containing complexes **51** and **53** by A549 cells was observed on fluorescence visualization under the confocal microscope (Figure 12). The preferential localization of complexes in mitochondria was found.

Thus, conjugates **52** and **53** containing distyryl-substituted BODIPY are highly efficient PDT agents. The observed activity of PDT is significantly higher than that of the hematoporphyrin preparation of PDT (Photofrin) approved by the PDT. Complex **53** meets all the basic requirements of PDT and is promising for further research aimed at potential therapeutic use.

### 3.2. BODIPY Conjugates with Carbohydrates

Carbohydrates are widely distributed in nature and play a very important role in human life, an integral component of the cells and tissues of a living organism. Properties, applications, and their benefits to humans confirm that these substances are the most important biological components. Many carbohydrates and their derivatives are medicine drugs. In this regard, the study of the processes of metabolism of mono- and polysaccharides is of great importance and can be carried out using conjugates with fluorophores.

**Scheme 31:**
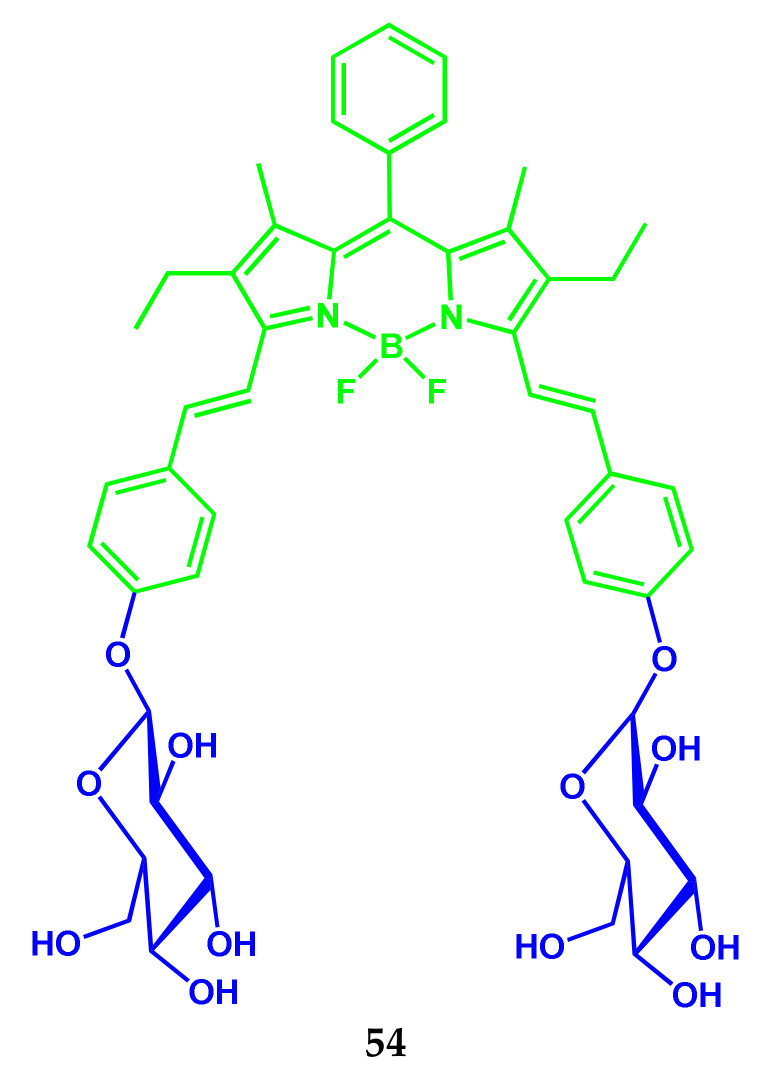
Structure of compound **54**.

Distyryl-substituted BODIPY **54** conjugated with glucose, which emits in the near IR range, was developed (Scheme 31) [49].

**Figure 13:**
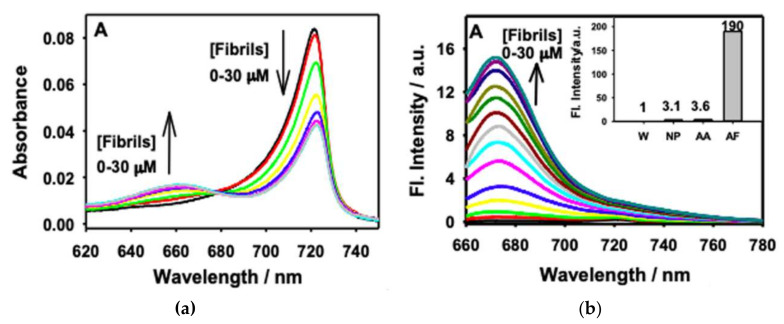
Changes in the absorption (**a**) and fluorescence (**b**) spectra of **54** (0.34 μM) upon addition of insulin fibrils (0–30 μM). Inset: Bar diagram for the change in emission intensity of **54** in water and different forms of insulin protein: W—water, NP—native protein, AA—amorphous aggregates, AF—amyloid fibrillar aggregates. Adapted from [49].

The probe shows a significant increase in fluorescence intensity with association with protein oligomers and ripened fibrils. When adding and increasing the concentration of fibrils, a significant decrease in absorption at 722 nm of conjugate **54** with a parallel increase in absorption at 660 nm were observed (Figure 13). The molecular form, that absorbs at 660 nm, is attributed to monomeric molecules of the conjugate, bound with insulin fibrils.

In this case, the fluorescence spectrum intensity of a slightly emitting **54** was increased 190 times at a wavelength of 670 nm. Therefore, the probe **54** has a great potential for visualization of protein oligomers and matured fibrils in vivo.

**Scheme 32:**
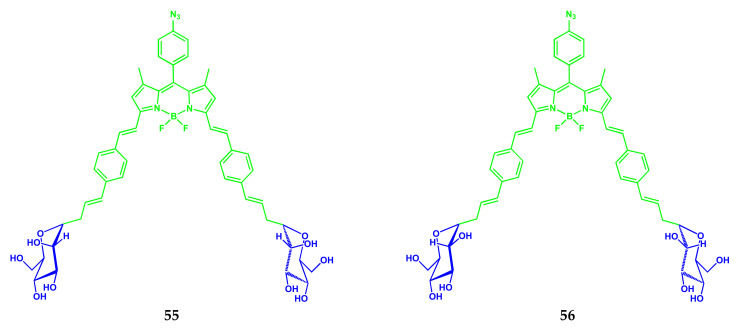
Structures of compounds **55** and **56**.

Giacomo Biagiotti and colleagues presented a synthesis and description of properties as well as studies for DC-SIGN super-resolution bioimaging of two Glyco-BODIPY conjugates: BODIPY-mannose **55** and BODIPY-glucose **56** (Scheme 32) [50].

Conjugates **55** and **56** are characterized by a high molar extinction coefficient of 43,746 ± 141 M^−1^ cm^−1^ (λ_max_ 652 nm) for **55** and 99,433 ± 204 M^−1^ cm^−1^ (λ_max_ 649 nm) for **56** in methanol. The fluorescence spectrum of **55** showed the maximum at 657 nm (λ_ex_ 580 nm), which is hypsochromic shifted to λ_em_ 654 nm with a decrease in concentration (from 0.78 μM and lower). Similar effects are observed for conjugate **56**. The relative fluorescence quantum yields (λ_ex_ 580 nm) of **55** and **56** were 0.24 and 0.17, respectively. In vitro cell studies using fluorescent microscopy proved that BODIPY-mannose conjugate **55** is effectively absorbed by immune cells, expressing the DC-SIGN receptor. Conjugate **55** was localized in endosomal membranes, which proves that **55** is a reliable tool also in stimulated emission depletion microscopy applications.

**Scheme 33:**
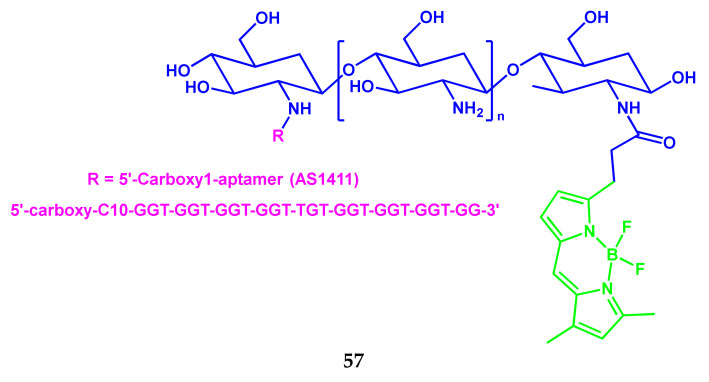
Structure of compound **57**.

In the study by Setareh Taki and Mehdi Shafiee Ardestani, the bio-conjugate **57** aptamer AS1411 with chitosan nanostructure and a fluorescent BODIPY-label was synthesized for use in breast cancer (T47D) diagnostics (Scheme 33) [51].

Based on the studies conducted by the authors [51], including the results of fluorescent visualization in vivo and flow cytometry in vitro, it was shown that the conjugate **57** can be used as a potential diagnostic agent for fluorescent visualization of cancer cells without toxicity for normal cells.

**Scheme 34:**
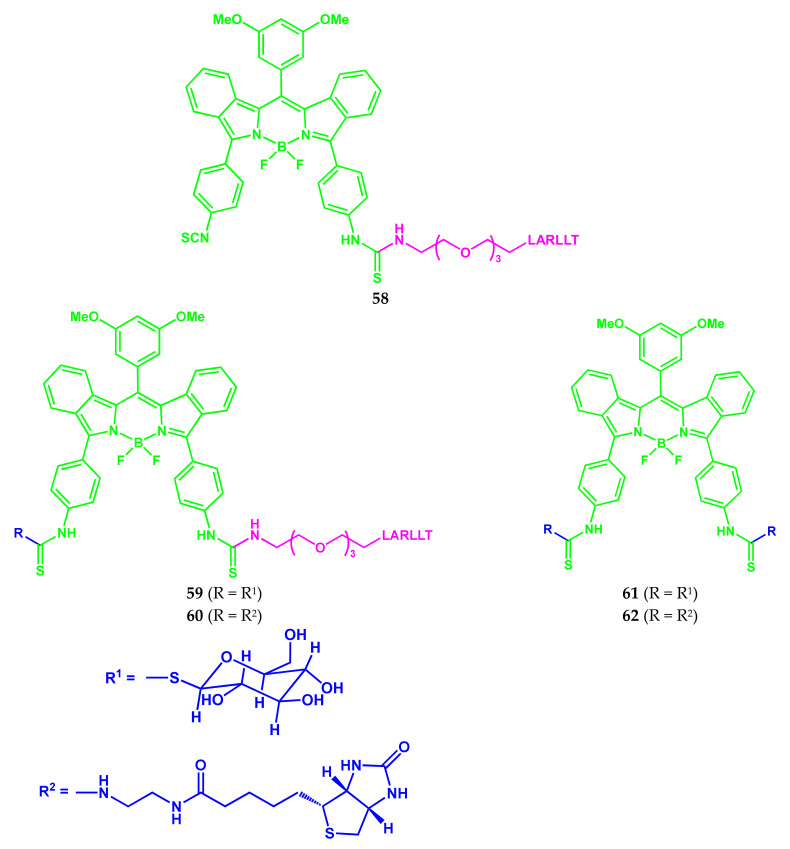
Structures of compounds **58**–**62**.

The five conjugates **58**–**62** based on NIR BODIPY containing an epidermal growth factor receptor (EGFR)-targeted pegylated LARLLT peptide and/or a glucose or biotin ethylene diamine group were synthesized (Scheme 34) [52]. Among them, three conjugates contained one 3PEG-LARLLT peptide and one group of water-soluble glucose or biotin (compounds **59** and **60**) or did not contain them (compound **58**). The other two conjugates were formed by BODIPY and glucose (**61**) or biotin (**62**) two fragments but did not contain a peptide.

All conjugates **58**–**62** showed absorption and fluorescence bands in the DMSO with maxima in the near IR ranges of 650–657 nm and 689–696 nm, respectively. The extinction molar coefficients were in the range from 13,700 to 72,600 M^−1^ cm^−1^, which is characteristic of BODIPY-peptide conjugates. All compounds showed a fluorescence quantum yield in the range of 0.37–0.47 in DMSO.

Cellular localization (colon cancer cells) and competitive binding studies showed that conjugates **59** and **60** with LARLLT peptide showed specific binding with the epidermal growth factor receptor (EGFR) protein, compared with the conjugates **61** and **62**, not containing peptide. Compounds **61** and **62** showed only nonspecific binding to EGFR. The results of extracellular localization (using fluorescent microscopy) showed that when using conjugates **59** and **60** in SW480 cells, a significantly higher fluorescence signal was observed compared to LOVO cells, which indicates that these compounds actively bind with EGFR. A weaker fluorescence signal was observed in the case of **61** and **62**, which indicates the non-specific binding of these compounds with cells. In general, all BODIPY conjugates showed low cytotoxicity, among which **61** is the least phototoxic.

The BODIPY-bio-conjugates containing a 3PEG-LARLLT-peptide are promising as agents for fluorescent visualization in the near-IR range for colon cancer, super-expressing EGFR.

### 3.3. BODIPY Conjugates with Lipids

Saponifiable lipid derivatives of sphingosine play an important role in life processes. Being one of the main components of biological membranes, sphingolipids affect their permeability, participate in the transmission of a nerve impulse, and the creation of intercellular contacts.

**Scheme 35:**
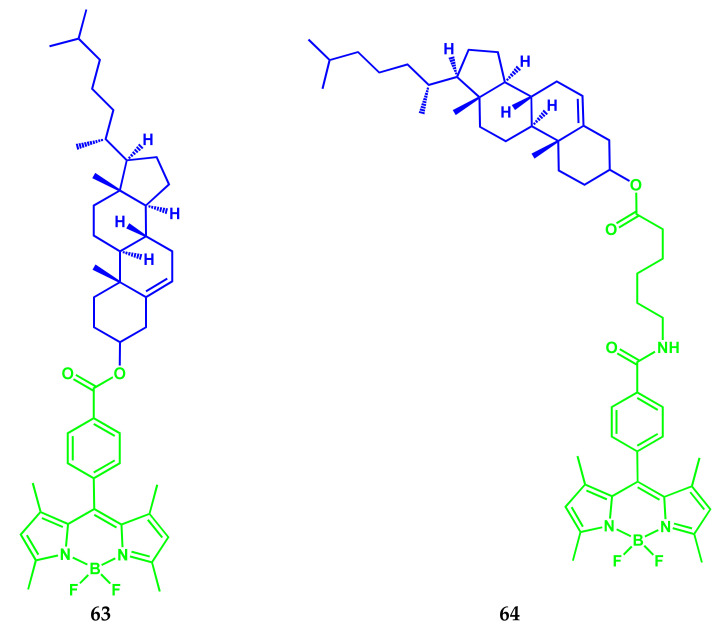
Structures of compounds **63** and **64**.

A simple method of conjugation of probe–cholesterol for the synthesis of **63** and **64** (Scheme 35), which differ in the length of the aliphatic chain between the dye and cholesterol, was described in [53]. For **63**, the probe and cholesterol were directly related to the complex–ester bond, and in the case of conjugate **64**, the probe was bound to cholesterol with a hexyl linker.

Conjugate **63** does not stain the membrane of living cells, permeates through the membrane, and is localized in lipid drops in HeLa and CHO cell lines. The **64** is held on the membrane of living cells, where he apparently stained the cholesterol-rich membranes.

**Scheme 36:**
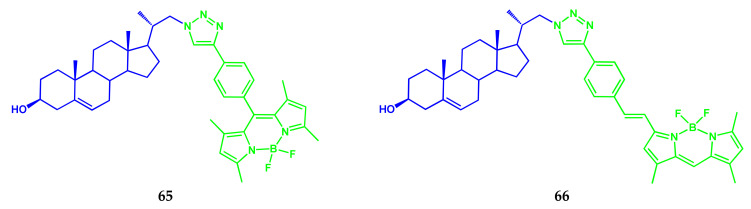
Structures of compounds **65** and **66**.

Zaiguo Li and Robert Bittman received BODIPY-conjugated cholesterol analogs **65** and **66** (Scheme 36) [54].

Compounds **65** and **66** demonstrate the absorption and emission spectra with maxima 499 and 569 nm, characteristic of the BODIPY-fluorophore with high extinction coefficients (110,000 and 80,000 M^−1^ cm^−1^, respectively) in ethanol and small Stokes shifts. The absorption and fluorescence maxima for conjugate **66** in chloroform are red shifted (on 8–9 nm) compared with ethanol, and the ε is 9000 M^−1^ cm^−1^ higher. Due to intensive absorption and fluorescence, conjugates **65** and **66** are promising as probes for monitoring cholesterol effects in model membranes and cells. Fluorescent properties of conjugates **65** and **66** allow visualizing the distribution and transport of lipids in living cells using fluorescent microscopy.

**Scheme 37:**
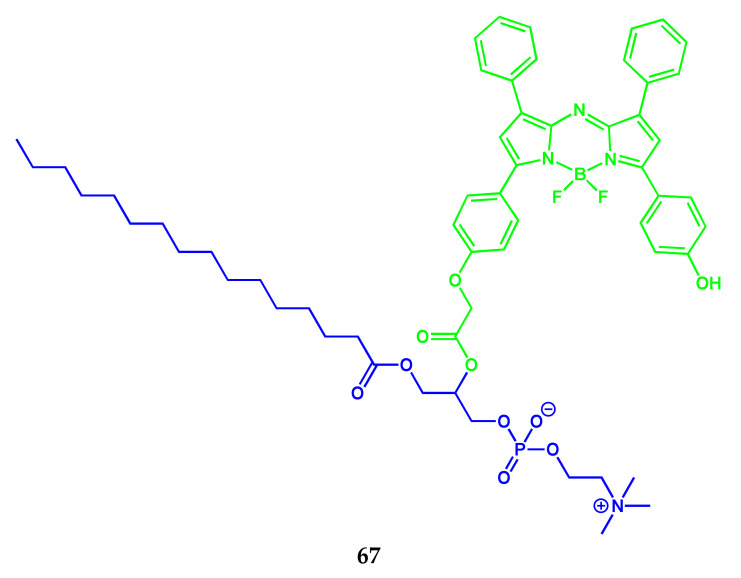
Structure of compound **67**.

**Figure 14:**
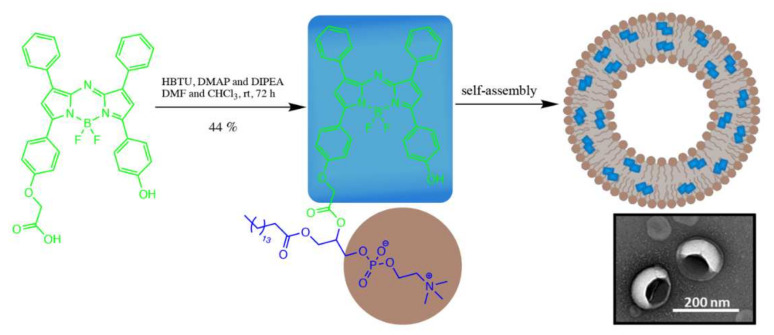
Self-assembly **67** into a BODIPYsome nanoparticle (phospholipid head group in brown and aza-BODIPY in blue). Adapted from [55].

The synthesis of the new building block of aza-BODIPY-lipid **67** (Scheme 37) and its self-assembly in the liposomal nanoparticle–BODIPYsome (BODIPY vesikul), capable of stable J-aggregation, were demonstrated in [55] (Figure 14).

Capabilities of conjugate **67** for optical visualization (photoacoustic/fluorescence) were observed on a mouse model of an orthotopic prostate tumor 24 h after intravenous. The authors note the prospect of BODIPYsome to develop new building blocks in the design of optically stable agents of biophotonic imaging.

**Scheme 38:**
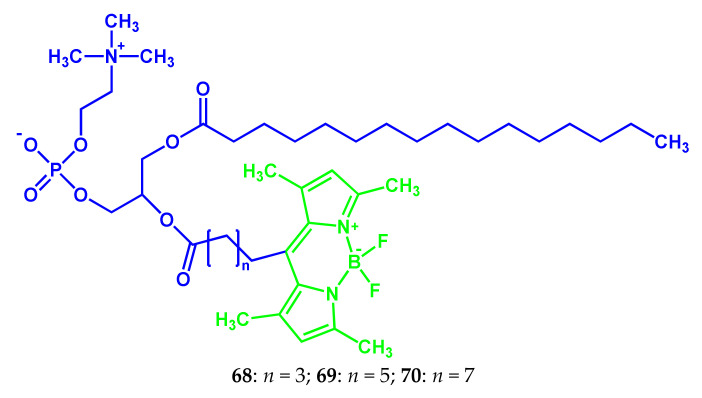

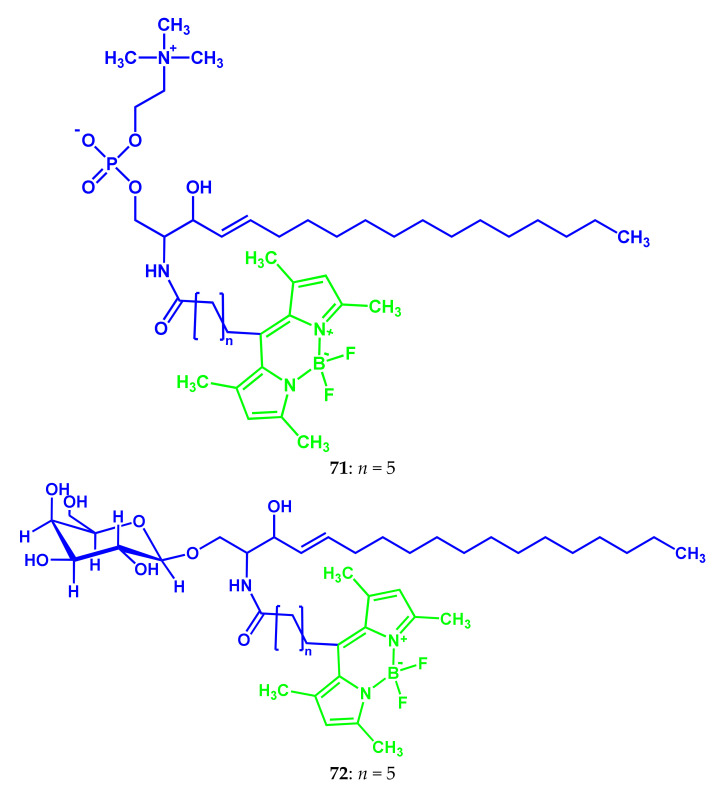
Structures of compounds **68**–**72**.

A series of BODIPY fluorescent probes: phosphatidylcholines **68**–**70**, sphingomyelin **71**, and galactosylceramide **72**, acylated by fatty acids of various chain lengths in the meso-position (Scheme 38), was synthesized and investigated in an article by Russian scientists [56].

All synthesized probes **68**–**72** have almost the same spectral properties: absorption bands with a maximum 498 nm (extinction coefficient is 80,000 M^−1^ cm^−1^) and fluorescence at 506 nm, typical for BODIPY derivatives. Probes can be easily included in artificial membranes (liposomes) by preparation of liposomes from the original lipid mixture containing a probe, and by adding probes to finished liposome drugs. The probe **71** was successfully used in a cytological study as a highly sensitive fluorescent dye for the specific staining of some organelles of blood neutrophils.

### 3.4. BODIPY Conjugates with Steroids

Steroids are widely distributed in nature and perform a variety of functions in biological systems. Sterane derivatives: vitamin D, glucocorticoids, mineralocorticoids, and sex hormones, are the most important regulators of life processes. Cholesterol, as a component of cell membranes, changes their fluidity.

**Scheme 39:**
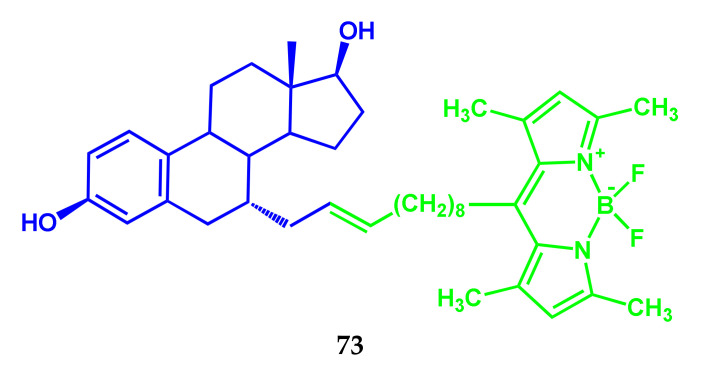
Structure of compound **73**.

Fluorescent BODIPY conjugate **73** with estradiol (E2) (Scheme 39) was obtained in [57]. The fluorescence quantum yield of conjugate E2-BODIPY **73** amounted to about 0.236 ± 0.071. Studies, conducted on rats subjected to ovariectomy, and non-ovariectomy mice, showed that E2-BODIPY stimulated the growth of the uterine tissue similar to the estradiol effect.

**Figure 15:**
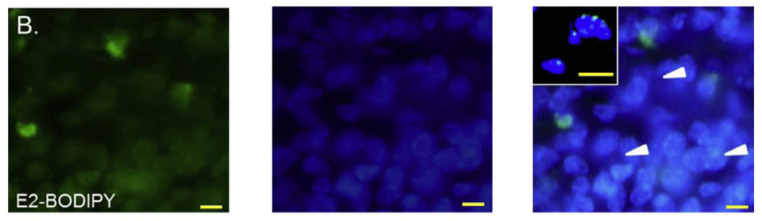
Subcellular localization of E2-BODIPY **73** in uteri of non-ovariectomized mice. The first image in each row is the emission fluorescence at 488 nm. The second image in each row is the image of the Hoechst 33,342 nuclear stain that has excitation/emission maxima at 350/461 nm when bound to DNA. The third image is a merged photo of the first two images. Row B shows intranuclear localization of E2-BODIPY. Adapted from [57].

The fluorescence of E2-BODIPY in the nuclei of the epithelial uterus cells was observed in mice without ovariectomy after subcutaneous injection of E2-BODIPY (after 24 h) (Figure 15).

Compound **73** has the properties of nuclear localization, which makes it useful for fluorescent visualization of E2-sensitive tissues in vivo.

**Scheme 40:**
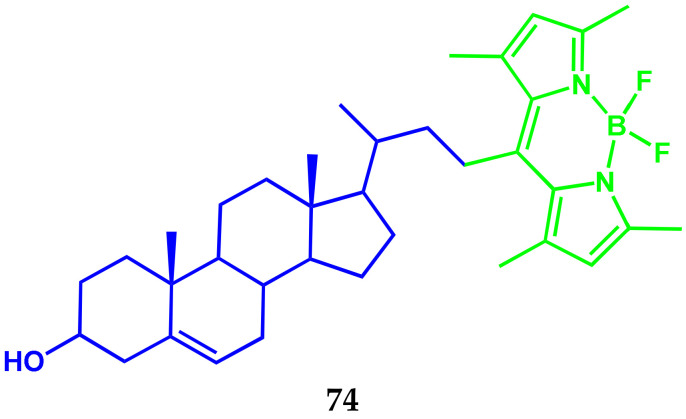
Structure of compound **74**.

The authors of [58] demonstrated the ability of steroids with fluorophores in the side chains to penetrate mycobacterial cells. Using fluorescent steroid **74** conjugated with BODIPY causes staining of both membrane lipids and cytosolic lipid drops during incubation (Scheme 40).

This makes it possible to create conjugates as molecular probes to study mycobacteria or the development of future anti-tuberculosis agents.

**Scheme 41:**
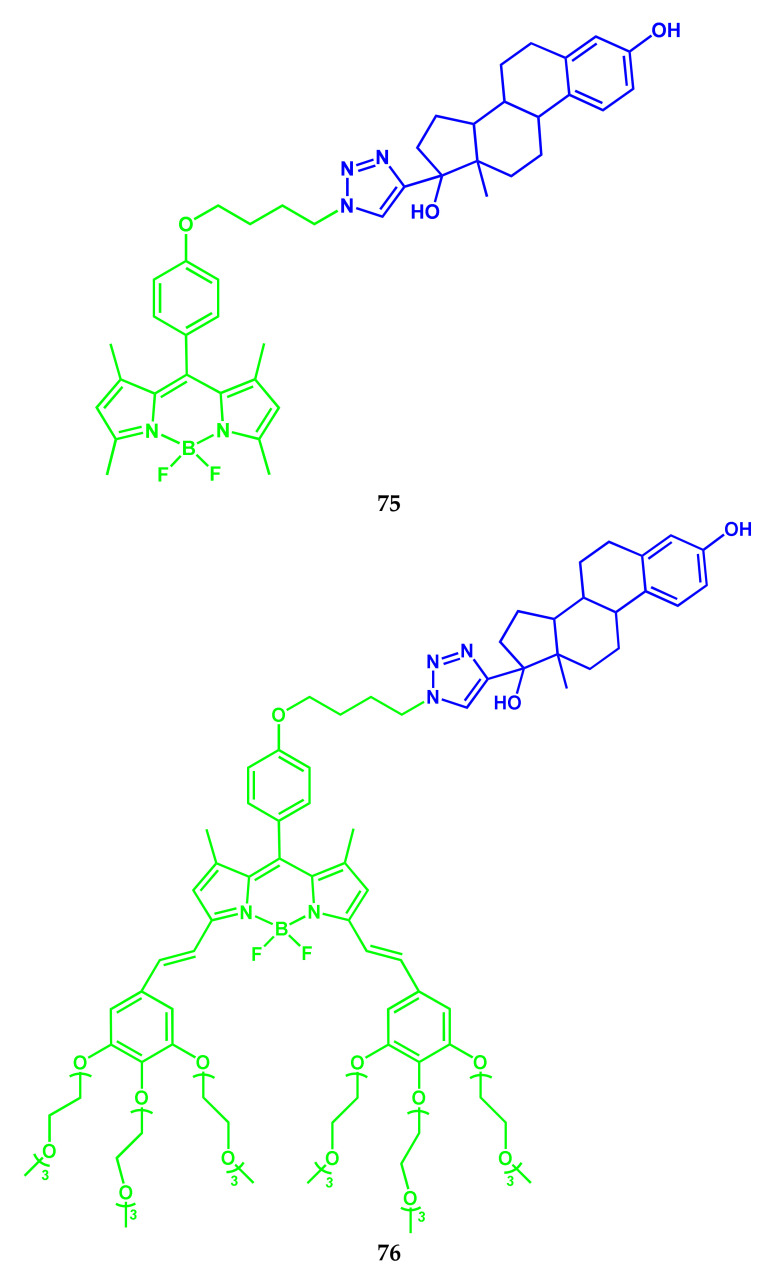
Structures of compounds **75** and **76**.

Two BODIPY conjugates with 17a-ethinylestradiol **75** and **76** (Scheme 41) were obtained as probes for fluorescent visualization at different wavelengths [59].

Conjugate **75** has an *S*^0^–*S*^1^ absorption band with a maximum of 500 nm and a molar extinction coefficient of 78,200 M^−1^ cm^−1^ in ethanol. The maximal absorption band of compound **76** was observed at 640 nm with ε = 41,100 M^−1^ cm^−1^. The fluorescence spectra of compounds **75** and **76** in ethanol contain bands with maxima at 508 and 650 nm and their fluorescence quantum yields are 0.45 and 0.27, respectively. Biovizualization studies have shown that compounds **75** and **76** are biologically functional and non-toxic for cells. The authors of [59] note, that conjugate **76** is more reactive compared with **75**, and is a water-soluble molecule, which increases bioavailability and, thus, causes the desired physicochemical properties. Water-soluble conjugate **76** is able to discriminate between breast cancer subtypes. The conjugate **76** may be an alternative to immunohistochemistry and eliminates the need for antibodies, that are unstable and expensive. The high molar absorption coefficient and fluorescence quantum yield of conjugates **75** and **76** satisfy the requirements of ideal probes for fluorescence visualization.

**Scheme 42:**
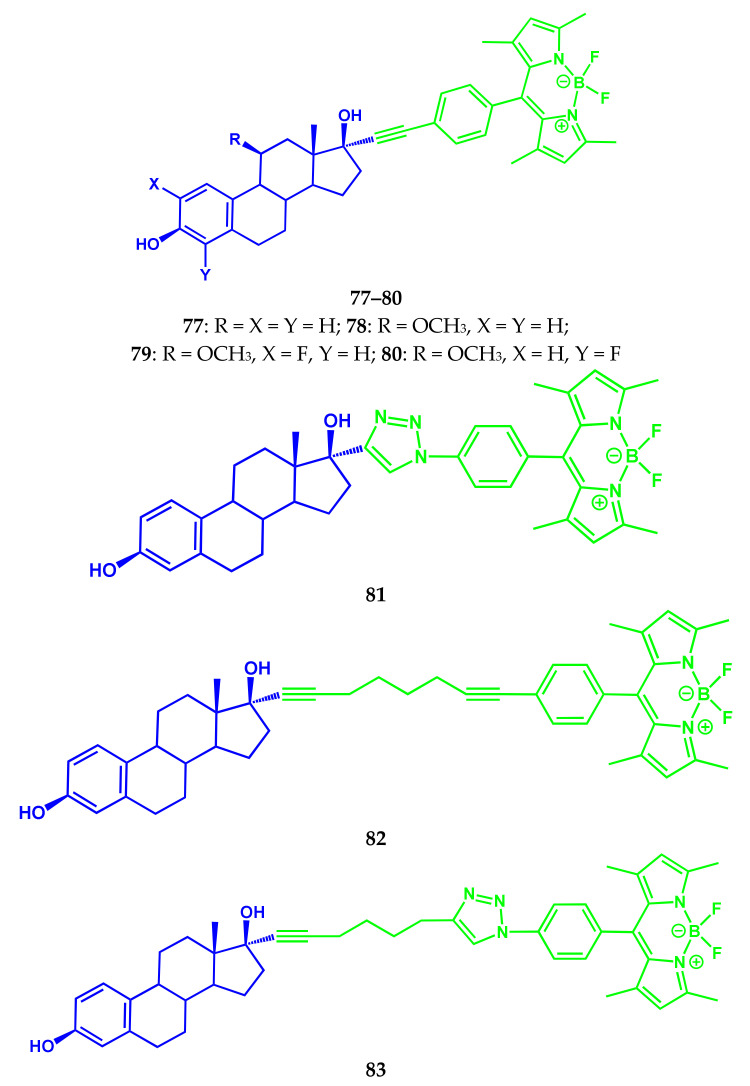
Structures of compounds **77**–**83**.

Studies of another series of BODIPY-estradiol conjugates **77**–**83** (Scheme 42), carried out by Samira Osati and colleagues in [60], were aimed at modifying both steroid fragments and a carbon spacer chain.

The absorption of conjugates **77**–**83** is characterized by a spectrum in the visible region with a maximum of the most intense long-wave *S*_o_–*S*_1_ band in the range 502–504 nm and the emission band maximum at 519–536 nm. In vitro studies allowed to establish the relative ERα binding affinity (RBA). The initial non-conjugated steroid EE2 showed a high binding affinity with ER (RBA = 170%). Conjugates BODIPY-EE2 **77**–**79** did not have an affinity for ER, while **80** showed a weak binding affinity with ER (RBA = 0.03%). The introduction of a linear eight carbon chain between EE2 and BODIPY fragments in a conjugate **82** with Erα leads to a binding affinity of 12.4–16.4%. In contrast, conjugate **83** also has a long spacer chain devoid of binding affinity with ERα, due to the polar nature of a triazine fragment, which can interfere with the binding process. The authors [60] note the need for further studies of conjugate **82** with high RBA values in vitro and in vivo for evaluating their potential for fluorescent visualization of ER for patient breast cancer.

### 3.5. BODIPY Conjugate with Human Epidermal Growth Factor Receptor-2

HER2 (human epidermal growth factor receptor 2) is a membrane protein, a tyrosine-protein kinase of the EGFR/ErbB epidermal growth factor receptor family, encoded by the human ERBB2 gene. Amplification or overexpression of the gene for this protein plays an important role in the pathogenesis and progression of certain aggressive types of breast cancer and is an important biomarker and therapeutic target for this type of cancer.

**Scheme 43:**
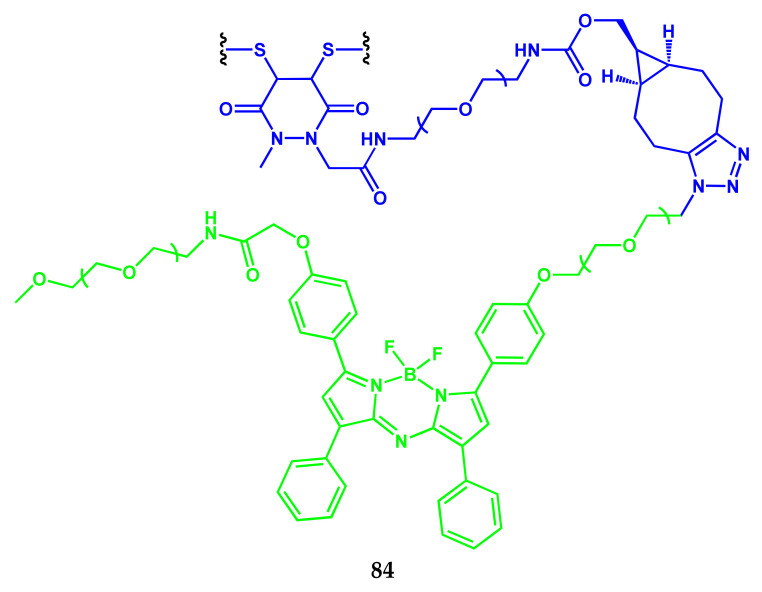
Structure of compound **84**.

Using a controlled and regioselective methodology of bioconjugation, the water-soluble and NIR-emissive conjugate **84** based on aza-BODIPY and trastuzumab (aza-BODIPY-trastuzumab conjugate) was synthesized and analyzed (Scheme 43) [61].

A study of the binding receptor of the cell surface using confocal laser scanning microscopy imaging in vitro demonstrated selectivity towards HER2 receptors. The results indicate the practical potential of aza-BODIPY-antibody conjugates as promising fluorescent NIR-probes for visualizing in vivo with promising applications in surgery under fluorescence control.

## 4. BODIPY Conjugates with Chromo- and Fluorophores as Diagnostic and Treatment Agents

Supramolecular systems with several chromophores are of growing interest to the scientific community due to their possible applications ranging from light-harvesting sensors and optoelectronic devices. The combination of two or more chromomorphic and/or luminophoric units in the structure of one molecule allows a variety of charge and energy transfer mechanisms to be implemented, which provide an excellent sensory release of the compound, or the manifestation of good therapeutic characteristics by it.

### 4.1. BODIPY Conjugates with Phthalocyanines

Being applicable as photosensitizers, phthalocyanines have received considerable attention as promising PDT agents. Although there are many examples of BODIPY and phthalocyanine compounds, only a few studies discussing their combination in the same supramolecule are known in the literature. Most of these molecules contain BODIPY groups as axial substituents on the boron subphthalocyanines or silicon phthalocyanines. BODIPY and PC conjugation can extend the absorption region and combine the advantageous characteristics of individual components. 

In the next review section, several examples of BODIPY-Pc conjugates are shown in the context of spectral and photophysical characteristics in comparison with the individual molecules and the opportunities of their practical application in molecular sensorics, medical diagnostics, and treatment.

There are several general synthetic routes for Pc-BODIPY conjugates, including axial coordination of BODIPY unit to Pc through the central metal atom, or direct chemical binding of Pc ligand’s peripheral groups with BODIPY.

#### Axially Coordinated BODIPY-Pc Conjugates

General synthetic routes for the BODIPY-Pc conjugates are usually as follows: phthalocyanine, BODIPY, and dry pyridine in dry toluene is refluxed for several days. After evaporation of the solvent in vacuum, the residue is dissolved and purified by preparative thin-layer chromatography on silica gel plates using CHCl_3_ as an eluent to give the desired product.

**Scheme 44:**
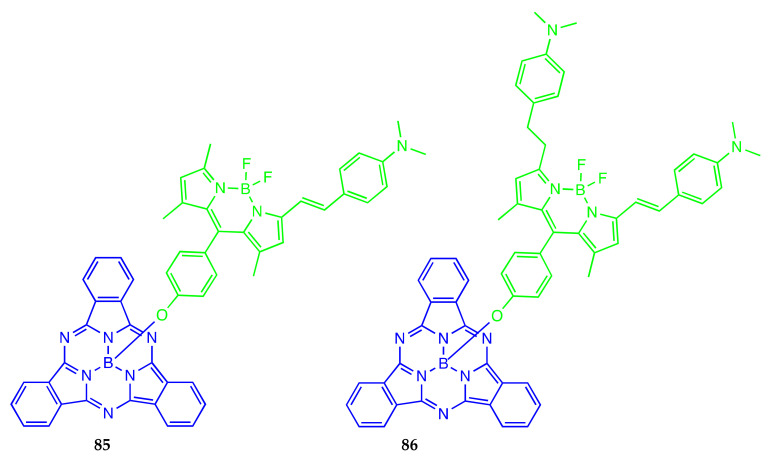

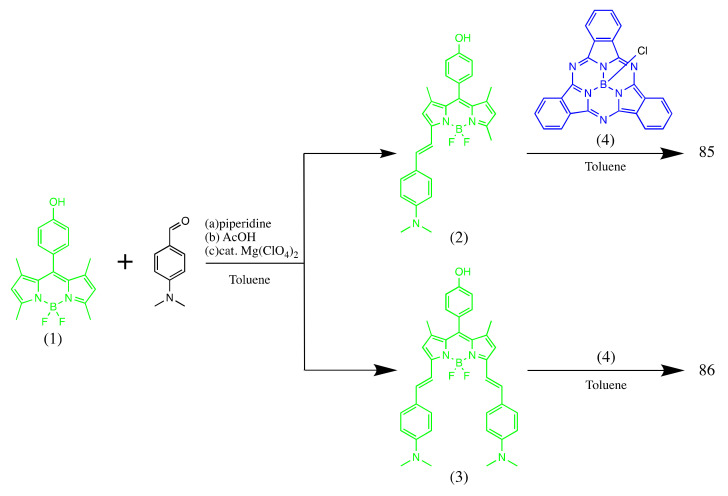
Structures and synthetic route for compounds **85** and **86**.

Boron(III) subphthalocyanines (SubPcs) are the lowest homologs of phthalocyanines bearing boron atoms within their central cavity. The combination of the bowl-shaped structure and delocalized 14π-electron aromatic conjugation system governs intense fluorescence and nonlinear optical properties for B(III)SubPcs. In the paper [62], the molecular design, synthesis, and characterization of BODIPY-subphthalocyanine dyes **85** and **86** were reported (Scheme 44).

The fluorescence peak related to the SubPc moiety (4) at 572 nm was reduced under the axial chlorine atom substitution with BODIPY groups to give **85** and **86**. This intensity reduction is caused by the FRET mechanism realization between BODIPY and SubPc. The emission spectra of compounds **85** and **86** were also compared with those of their starting compounds (2) and (4) under the same conditions. The significant quenching of donor emission and an enhancement in acceptor emission were observed for **85** and **86**. Compound **85** showed a more pronounced energy transfer in comparison with **86** because of the higher overlap of the donor-acceptor bands in the corresponding spectra.

The dyes **85** and **86** showed higher singlet oxygen generation compared to their parent compounds.

**Scheme 45:**
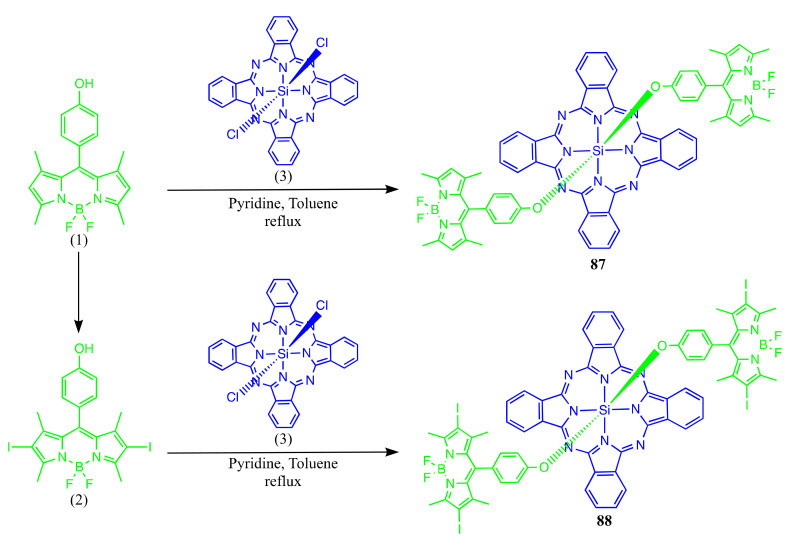
Structures and synthetic route for compounds **87** and **88**.

The article [63] describes a novel Si(IV)Pc dye substituted with iodinated-BODIPY groups (Scheme 45). The authors showed that there are no spectral changes in the absorption wavelengths after substitution.

**Figure 16:**
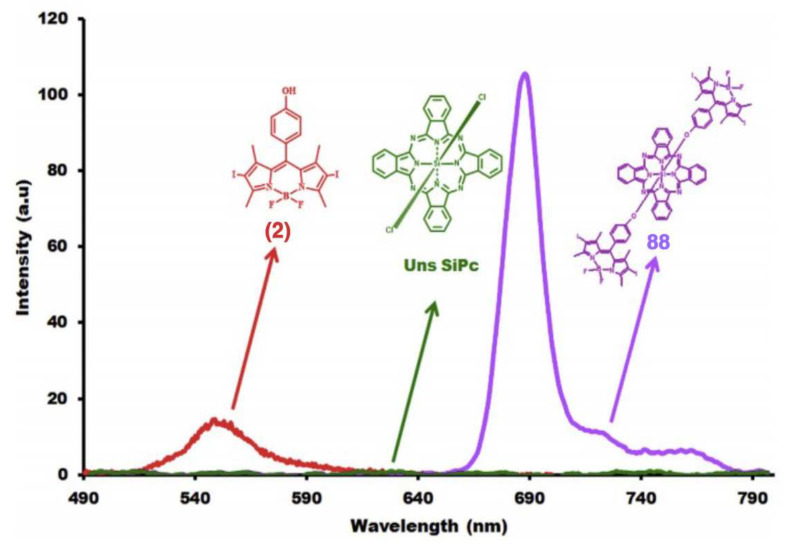
Emission spectra of compounds (2), **88** and unsubstituted silicon(IV) phthalocyanine in toluene. Adapted from [63].

Pc’s have a strong tendency to aggregate and negatively influence spectral and photophysical characteristics. Compound **88**, being the Pc-BODIPY conjugate, did not show any aggregation in all studied solvents with a narrow Q band observed at around 680 nm (Figure 16). Iodo-BODIPY caused a lowering of the Φ_F_ value being 0.12 for **88** in toluene in comparison with non-iodinated BODIPY-Si(IV)Pc **87** (Φ_F_ = 0.60) due to the heavy atom effect. According to the equation, the energy transfer quantum yield value of phthalocyanine **88** was observed (0.95), and the singlet oxygen generation yield value (0.46) characterizes **88** as a potential photosensitizer in PDT applications.

**Scheme 46:**
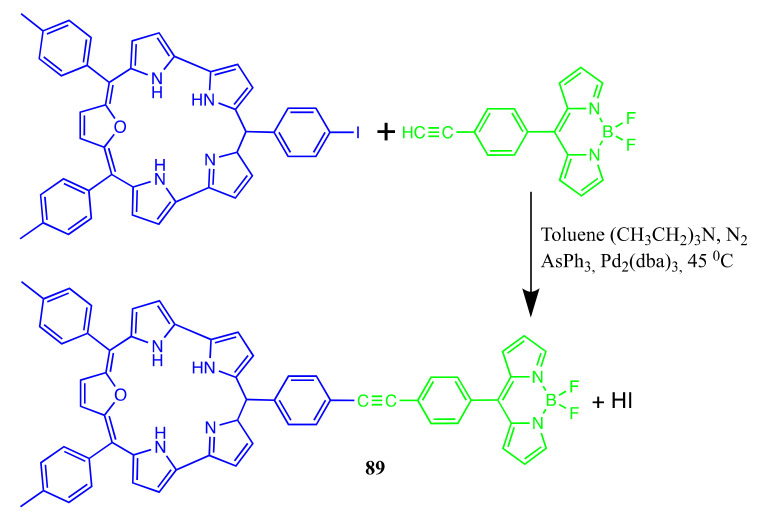
Structure and synthetic route for compound **89**.

Recent studies have shown the importance of oxasmaragdyrin sensitizer as a compelling material for light-energy-harvesting applications [64]. The coupling of oxasmaragdyrins with BODIPY enhances the photosensitization of **89** (Scheme 46).

**Scheme 47:**
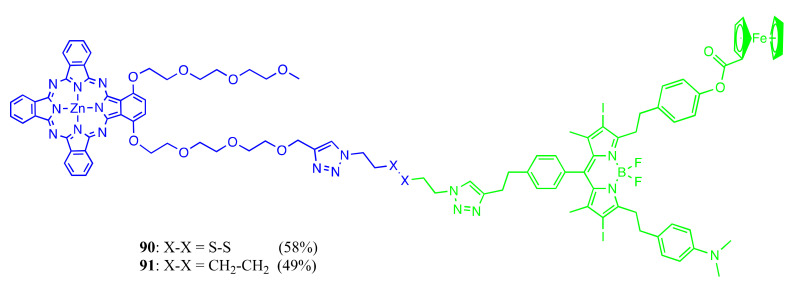
Structures of compounds **90** and **91**.

FRET-based activatable perspective photosensitizers **90** and **91** (Scheme 47) with near-IR absorption were obtained based on ferrocenyl BODIPY [65].

**Scheme 48:**
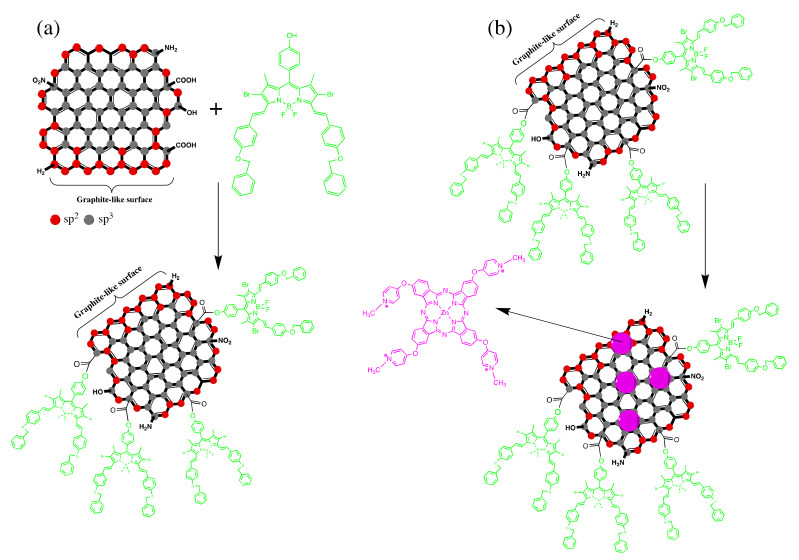
Structures and synthetic route for the hybrid material of DND with BODIPY (**a**) and combined with ZnTPPcQ (**b**).

A nanocomposite consisting of Pc, BODIPY, and DNDs with the aim of improving PDT activity through synergistic effect was synthesized [66]. The composites were constructed under the covalent linking of the DNDs carboxylic groups and the hydroxyl moiety of the BODIPY, (ZnTPPcQ) was then adsorbed onto DNDs-BODIPY via π–π stacking interaction (Scheme 48).

**Figure 17:**
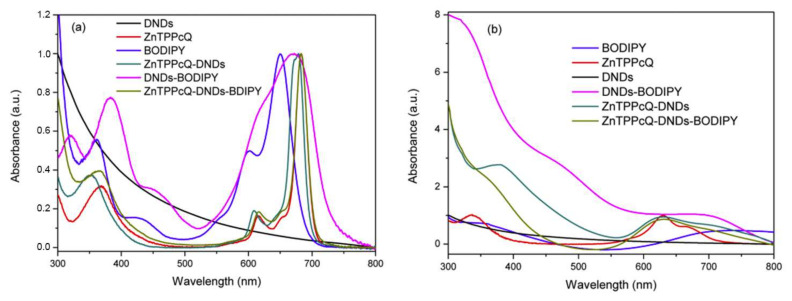
UV–Vis spectra of DNDs, ZnTPPcQ, BODIPY, DNDs-BODIPY and ZnTPPcQ-DND, and ZnTPPcQ-DNDs-BODIPY in (**a**) DMSO and (**b**) water (containing 1% DMSO) for all samples. Adapted from [66].

BODIPY spectral peak is red shifted upon conjugation to DNDs due to aggregation (Figure 17). DND also decreases Φ_F_ and τ_F_ of photosensitizers. The excitation wavelength at 649 nm excites both the BODIPY and Pcs, and the aforementioned Φ_F_ and τ_F_ values are influenced by both photosensitizers.

**Scheme 49:**
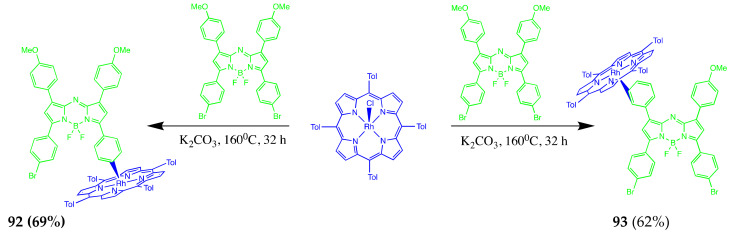
Structures and synthetic route for compounds **92** and **93**.

Remarkably, photostable NIR-absorbing and -emitting dyes **92** and **93** have been synthesized (Scheme 49) of a Group 9 metal porphyrin with an aza-BODIPY [67]. The BODIPY moiety can be easily modified to deal with the impact on the spectral properties. To take advantage of both types of compounds, an orthogonal conjugate with a direct metal-C bond should be obtained.

**Scheme 50:**
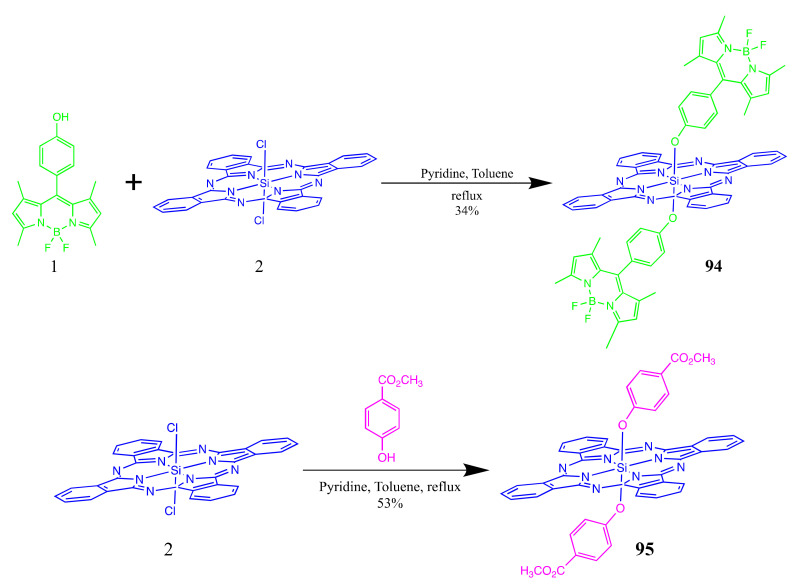
Structures and synthetic route for compounds **94** and **95**.

Si(IV)-based Pc’s were used for axial BODIPY coordination **94** [68]. Conjugate **95** was used as a reference (Scheme 50).

Absorption in Toluene, when excited at 480 nm, is observed only for BODIPY fragments, and triad **94** gave a radiation band at 687 nm due to the phthalocyanine nucleus. At a wavelength of about 510 nm, BODIPY radiation was not observed. The excitation of the reference compound **95** for a similar position did not give a radiation band at 687 nm. Based on this, we can discuss the emergence of an effective photoinduced energy transfer from excited BODIPY units to the phthalocyanine nucleus in **94**. The corresponding spectra for **95** and the equimolar mixture and **95** did not show a band characteristic of BODIPY at a wavelength of about 500 nm, but a band characteristic of phthalocyanine at 680 nm was observed. The quantum energy transfer yield (FENT) was estimated at 57%.

**Scheme 51:**
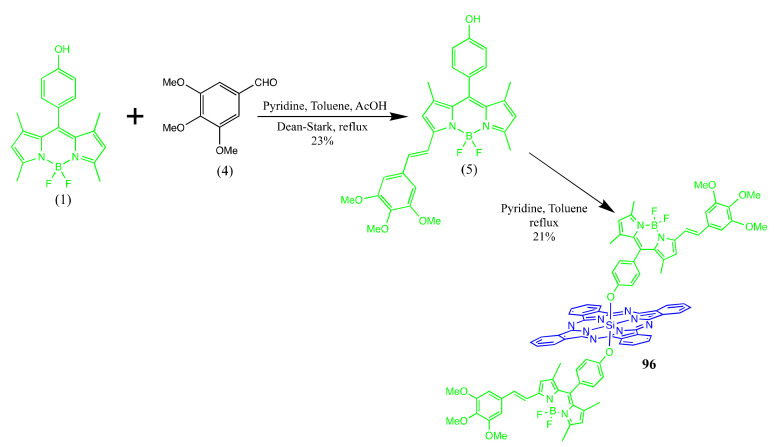
Structure and synthetic route for compound **96**.

In toluene, the singlet excited states of both reference compounds (1) and **95** decayed monoexponentially with a lifetime of 3.03 ± 0.01 and 5.07 ± 0.01 ns, respectively. For triad **94**, phthalocyanine fluorescence attenuation was detected when the fragment was excited-BODIPY, which also occurred in a monoexponential manner with a lifetime of 5.20 ± 0.01 ns. From the data obtained, it was concluded that electron transfer did not occur in **94** to suppress excited phthalocyanine. The quantum yield of fluorescence of phthalocyanine radiation, when excited by BODIPY fragments, was much lower compared to the direct excitation of the phthalocyanine nucleus. Moreover, electron transfer is a good way to quench excited BODIPY in **94**, on par with energy transfer. With two elongated monostyryl substituents, BODIPY triad **96** acts completely differently (Scheme 51).

After excitation of BODIPY **96** monostyryl fragments at a wavelength of 530 nm, BODIPY radiation with a wavelength of about 580 nm significantly decreased compared to radiation (5). The values of the fluorescence quantum yield decreased from 0.78 (for (5)) to 0.003 (for **96**). A very weak radiation band at 686 nm was also observed, which was stronger than that of **95** excited in the same position. The transfer of excitation energy from the initially excited monostyryl fragments of BODIPY to the phthalocyanine nucleus occurred in **96**, but the emission of fluorescence was largely suppressed. Since the photoinduced transfer of excitation energy from the initially excited phthalocyanine to the monostyryl BODIPY block is energetically unprofitable, quenching is expected to occur primarily due to the electron transfer process. Transition absorption spectra of **96** in toluene were also recorded when excited at 530 and 615 nm. The two spectra were very similar, showing a strong change in the color of the phthalocyanine and monostyryl bands in the BODIPY ground state at wavelengths of about 680 and 570 nm, respectively. In addition, a new band with a wavelength of about 580 nm appeared, which can be attributed to the phthalocyanine radical anion.

**Scheme 52:**
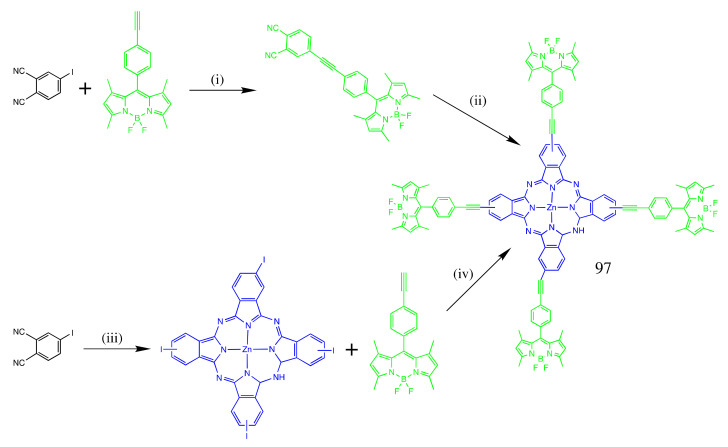
Structure and synthetic route for compound **97**.

A new type of pentadiene phthalocyanine **97** containing four blocks of boron dipyrromethene (BODIPY) at the peripheral positions of the phthalocyanine skeleton was developed and synthesized for the first time [69] (Scheme 52). 

Two different synthesis strategies were used to synthesize the target dye BODIPY-Zn(II)Pc pentade. The base-catalyzed cyclotetramerization of substituted phthalonitrile BODIPY (BODIPY-Pht), which is commonly used for the synthesis of phthalocyanine compounds, was the first one with the yield (6%). Another strategy is to synthesize a new target BODIPY-Pc through a Pd-catalyzed Sonogashir binding reaction between ethyl-BODIPY and iodine-Pc. Synthesis of the desired new BODIPY-Pc was achieved with a high yield (83%) using this compound reaction. Phthalocyanine compounds almost never dissolve in most organic solvents; however, the introduction of BODIPY substituents into the phthalocyanine ring increased the solubility, and the target BODIPY-Pc showed excellent solubility in the most common organic solvents (DMSO, acetone, ethyl acetate, DMF, toluene, benzene, chloroform, and THF). The electronic spectrum of the BODIPY-Pc shows a panchromatic behavior that appears in the absorption over a broad spectral region and is able to use an efficient light-harvesting system. This spectrum contains three main characteristic bands related to the phthalocyanine core (698 nm and 370 nm) and a BODIPY unit (502 nm). The observed single (narrow) Q band at 698 nm is typical for non-aggregated metallophthalocyanine complexes. The effect of the BODIPY substitution on the phthalocyanine skeleton is also clearly apparent on inspection of the UV–Vis absorption spectrum of this pentad dye. The Q band-based phthalocyanine unit is 18 nm red-shifted when substituted with BODIPY groups. This observed red-shifted Q band indicates that two chromophores (BODIPY and Pc) possess significant ground-state electronic interactions. BODIPY fluorescence band at 516 nm virtually decreased when excited at the same excitation wavelength for BODIPY-Pc. Instead, an additional emission band was observed at 710 nm, which is due to the phthalocyanine part of this dye. The Iodo-Pc compound did not show any emission band when excited at 480 nm at the same concentration because this compound does not show any absorption in this region. An efficient singlet–singlet energy transfer process in BODIPY-Pc from the excited BODIPY part to the phthalocyanine unit occurred. It is concluded that the Dexter model which occurs through covalent bonds is the mechanism responsible for energy transfer from donor BODIPY units to the acceptor phthalocyanine core. The fluorescence quantum yield (Φ_F_) for Ethynyl-BODIPY in THF is 0.55, while that for the BODIPY part of BODIPY-Pc is greatly reduced to 0. The ΦENT value was found to be 0.86, showing that this is an efficient energy transfer process for BODIPY-Pc. The singlet oxygen generation quantum yield ΦΔ value of BODIPY-Pc was estimated as 0.69 in DMSO which is higher than that of the starting Iodo-Pc compound (ΦΔ = 0). The substitution of iodine atoms with BODIPY units on the phthalocyanine framework has led to an increase in the singlet oxygen generation efficiency which might be derived from the energy transfer from BODIPY units to the phthalocyanine moiety.

**Scheme 53:**
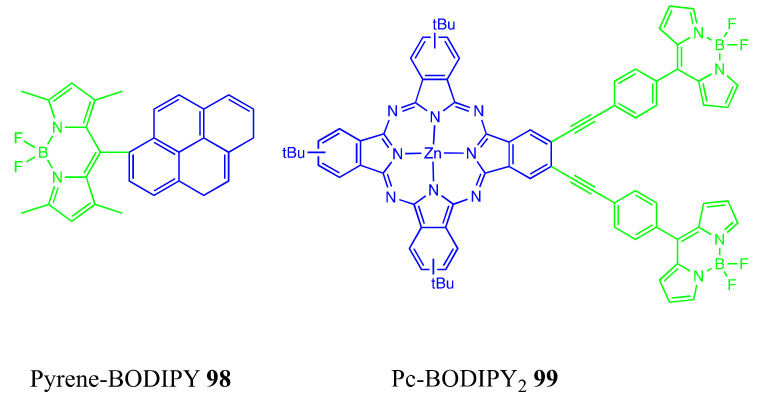

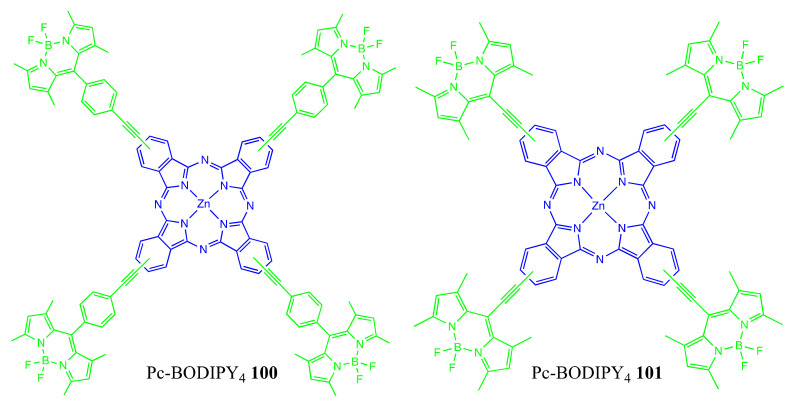
Structures of compounds **98**, **99**, **100**, **101**.

The syntheses of several BODIPY-Pc fluorophores conjugates and subsequent cellular imaging upon irradiation in the 300–500 nm window with a resulting emission beyond 500 nm and up to 720 nm was presented in paper [70]. From a synthetic standpoint, the conjugation of up to four BODIPY per Pc platform was difficult to achieve: a mixture of BODIPY-Pc conjugates, consisting of 2.5 BODIPY per Pc platform was obtained instead. The probes are not soluble in water; hence, they were formulated in DMSO (1% vol. in RPMI) or in liposomes. The study’s proof-of-concept was met, all probes **98**–**101** led to efficient staining of cells (Scheme 53), with good contrast and dark background, with staining of membranes and cytoplasm. Spectrofluorimetry studies showed that upon excitation of the BODIPY antenna (in the BODIPY-Pc conjugate), the cascade energy transfer to the Pc acceptor appeared to be complete because no subsequent emission of the BODIPY antenna occurred, whereas a strong emission of the Pc moiety was observed. However, biphoton microscopy still shows intense BODIPY emission in the green canal upon excitation at 750–800 nm (ca. 400–450 nm).

Upon excitation at 551 nm (BODIPY), subsequent fluorescence emission spectra showed that the transfer from the BODIPY antenna towards the Pc platform occurred. Indeed, the emission peak corresponding to the maximum emission of Pc was observed (700 nm). Moreover, when the excitation was achieved at 373 nm subsequent fluorescence emission also occurred leading to a similar emission spectrum.

Cell imaging studies were carried out in light of previous reports using BODIPY, Pc monomeric probes. Staining of membranes and cytoplasm occurred, whereas the nucleus remains unstained. This is unlike cells treated with Pc-BODIPY conjugate **101** in RPMI solution with 1% DMSO: strong fluorescence emission was observed mostly in the green canal but also in the blue and red canal. It indicates that cells were stained with fluorescent probes **101**, results with different canals also indicate that transfers from one antenna to the other occurred and may be reminiscent of the spectrofluorimetry studies. Altogether, microscopy and spectrofluorimetry studies seem to indicate that transfers from the donor to the acceptor moiety within a fluorophore conjugate do occur.

**Scheme 54:**
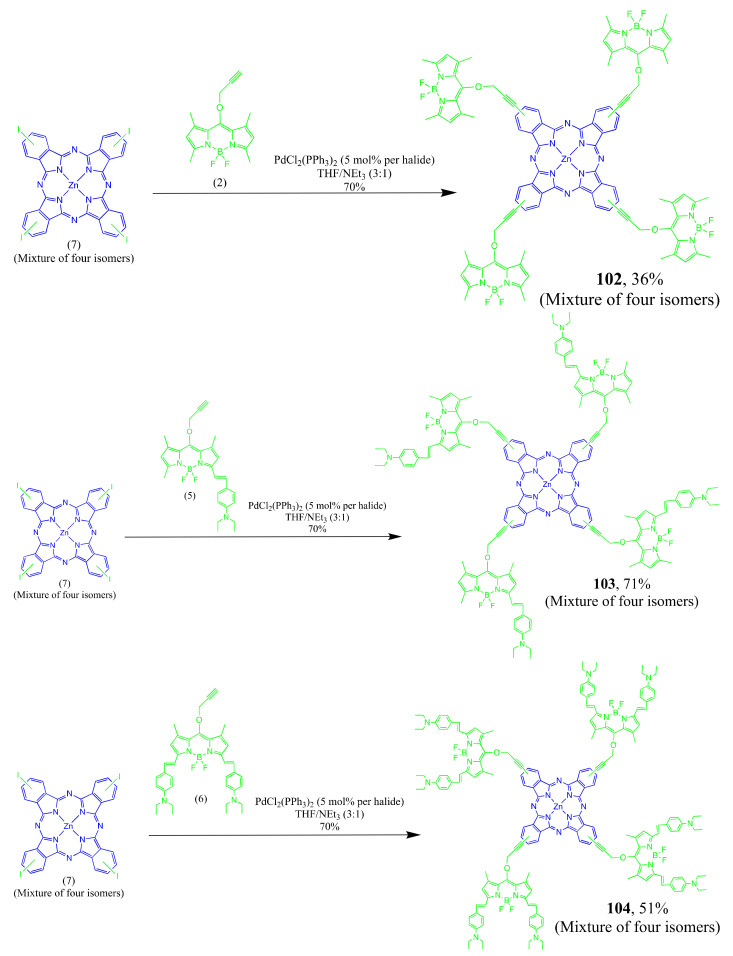
Structures and synthetic route for compounds **102**, **103**, **104**.

The examples of the new zinc(II) phthalocyanines containing BODIPY **102**, its monasteries (**103**) and distiryl (**104**) derivatives with p-(N,N-diethylamino) benzaldehyde fragments were synthesized [71]. These conjugates **102**–**104** were synthesized during the reaction of a copper-free Sonogashira compound of terminal ethinylfunctionalized BODIPYs with tetrakis(iodo) zinc(II) phthalocyanine (Scheme 54).

By replacing the p-(N,N-diethylamino) benzaldehyde groups, the electron density of the BODIPY system changes, as a result of which the maximum absorption wavelengths of the BODIPY groups (2), (5), and (6) are different in their ground state absorption spectra. The maximum absorption bands were observed at 500, 620, and 719 nm for (2), (5), and (6), respectively. Characteristic absorption bands were observed for both BODIPY fragments and phthalocyanine in the absorption spectra of the ground state of conjugates **102, 103,** and **104**. The absorption bands belonging to the BODIPY groups are shifted to longer wavelengths due to the replacement of p-(N,N-diethylamino) benzaldehyde groups at position 5 only for conjugate **103**, or both 5 and 7 positions for conjugate **104**, however, the characteristic Q and Soret bands (B bands) belonging to the phthalocyanine nucleus are almost not shifted and were observed at 690 and 350 nm for each conjugate. For zinc(II)-BODIPY phthalocyanine conjugates **102**–**104**, absorption bands characteristic of BODIPY particles were observed at 500 nm for **102**, 622 nm for **103**, and 719 nm for **104**.

When excited by the phthalocyanine motif at 640 nm, compound **102** had a maximum peak of fluorescence emission at 700 nm. However, when excited by the BODIPY motif at 480 nm, two radiation peaks are observed at 515 and 700 nm. A decrease in the radiation peak at 515 nm and the formation of a radiation peak at 710 nm were observed when excited at 480 nm in the radiation spectrum of conjugate **102**. This is due to the singlet-singlet energy transfer from the BODIPY groups to the phthalocyanine core, since no radiation was observed for unsubstituted zinc(II) phthalocyanine when excited at 480 nm. Based on the above, it can be argued that the mechanism of the Förster model, occurring through covalent bonds, is responsible for the transfer of energy from donor BODIPY units to the acceptor phthalocyanine nucleus. For the studied conjugates **103** and **104** (styryl BODIPY substituted zinc(II) phthalocyanines), very low fluorescence emission spectra were observed when excited at 550 and 650 nm, respectively. For these conjugates in acidic media, an increase in fluorescence intensity was observed due to similar effects described earlier. For conjugates **103** and **104**, an increase in fluorescence emission in acidic media is very important for use in PDT. An achievable parameter for the use of these compounds in the therapeutic field is the difference in pH between cancerous tissue and normal tissue. It follows from this that these photosensitizers can be used to diagnose cancerous tissues due to their fluorescence on-off properties.

It was found that ΦΔ for conjugate **102** is 0.56, which is equal to the value for unsubstituted ZnPc in DMF. The value of ΦΔ for this conjugate was higher than that of conjugates **103** and **104** (ΦΔ = 0.21 for **103** and ΦΔ = 0.07 for **104**). This can be attributed to the self-extinguishing of these conjugates, since the absorption and emission bands of both phthalocyanine fragments and BODIPY were very close to each other. More specifically, the BODIPY part, when interacting with light, can absorb the energy received from the phthalocyanine fragment, or the phthalocyanine frame itself can absorb the energy emitted by the BODIPY fragments. We conclude that the BODIPY and phthalocyanine groups act as a donor and acceptor at the same time, therefore, due to photoinduced electron transfer (PET), singlet oxygen is generated.

### 4.2. BODIPY Conjugates with Coumarin

Coumarin dyes are often used to provide contrast in multicolor applications, but coumarin fluorescence may be obscured by autofluorescence, they are only recommended for use with highly abundant targets or in cell tracking applications. The use of a combination of coumarin-BODIPY fluorophores could be promising due to the fluorescence of the former being close to UV and the fluorescence of the latter being more bathochromic. This combination allows one to expect the implementation of the mechanisms of charge and energy transfer, which contributes to an increase in the selectivity of the implementation of the response to a change in the molecular environment.

**Scheme 55:**
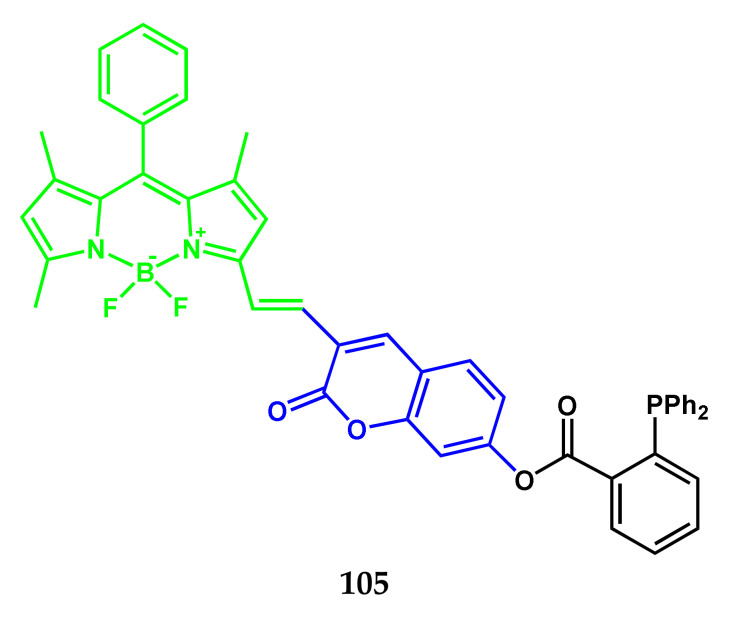
Structure of compound **105**.

Highly sensitive and selective ratiometric fluorescent probe **105** (Scheme 55) of the sensitive HNO recognition site based on the BODIPY-coumarin platform and triarylphosphine was demonstrated in [72].

The probe exhibits a significant shift in emission (up to 129 nm), low cytotoxicity, and good permeability of the cell membrane and is suitable for fluorescent ratiometric HNO visualization in living cells.

### 4.3. BODIPY Conjugates with Nile Red

Nile red (also known as Nile blue oxazone) stains intracellular lipid droplets. Nile red fluorescence is dependent on the medium polarity, being nonfluorescent in polar solvents, deep red fluorescent in polar membrane lipid, and strongly yellow-gold fluorescent in neutral lipid in intracellular storages. High solvatochromaticity makes this dye promising as the component for functional multichromophoric tools. 

**Scheme 56:**
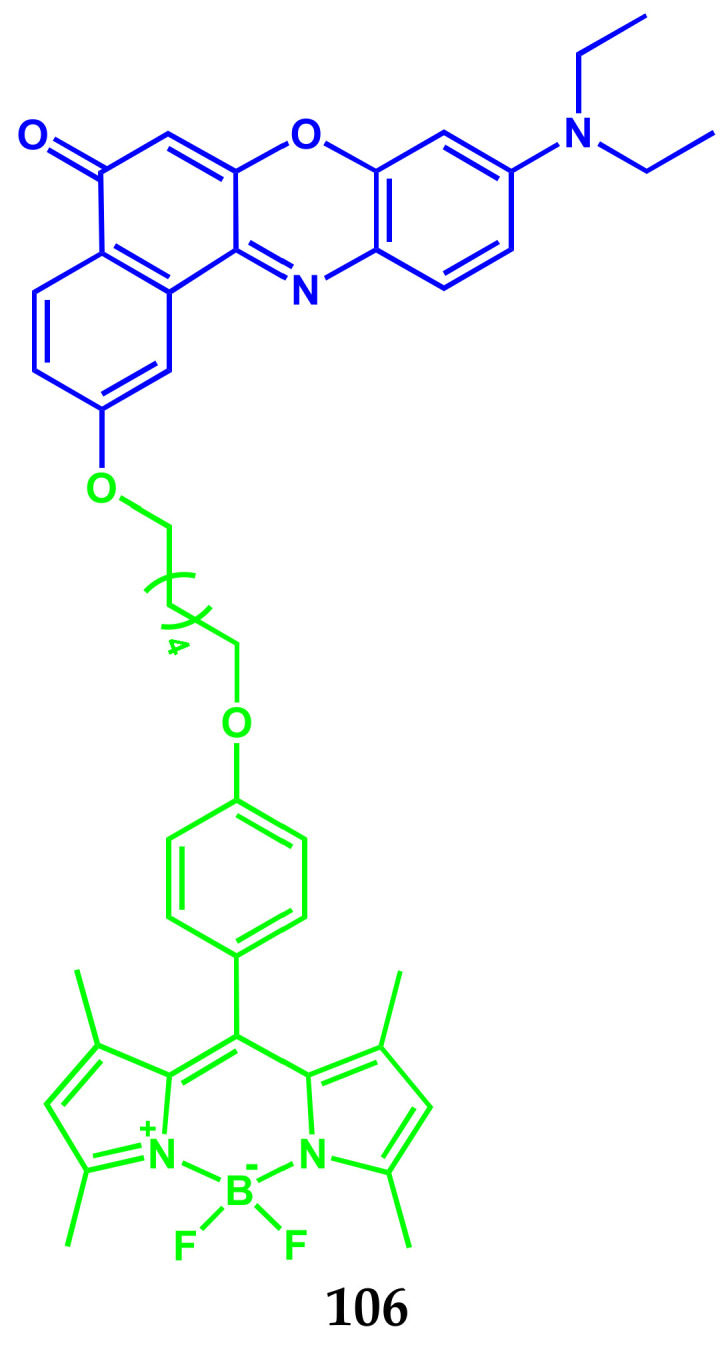
Structure of compound **106**.

A bimodal fluorescent Nile Red-BODIPY probe **106** sensitive to the microenvironment for the simultaneous detection of the local polarity and viscosity of endoplasmic reticulum in living cells was synthesized [73] (Scheme 56).

The probe **106** is localized in the endoplasmic reticulum, shows low cytotoxicity, and can be used to quantify various changes in both local viscosity and local polarity of ER-stressed HeLa cells.

## 5. Conclusions

Due to the high quantum yield of fluorescence, narrow and intense radiation profiles in the region of the phototherapeutic window, stability in solutions, and solid phase, non-toxicity of BODIPY, it is used in various fields of science and technology. The results of studies in the field of using BODIPY luminophores as markers of molecules of medicinal and biologically active compounds indicate great prospects for the use of luminophores of this class in medicine and biochemistry. The most promising areas include, first, the development of BOIDPY conjugates with drug, in which the luminophore, along with the functions of a biomarker, exhibits a therapeutic effect. For example, such fluorescent drug conjugates are capable of being selectively recognized and absorbed by cancer cells, in which, under the influence of light, they are degraded to an active cytotoxic drug and BODIPY, which acts as a PDT agent.

Equally interesting is the conjugation of BODIPY with molecules of biologically active substances, which makes it possible to track the processes of their transport, localization in cell organelles, and metabolism.

## Figures and Tables

**Table 1:** Practically significant characteristics of BODIPY conjugates with platinum drugs.

Conjugate	*λ*_abs_, nm (*ε*, M^−1^ cm^−1^)	*λ*_fl_, nm	Φ_F_	ΦΔ	Comments	IC_50_, μM	Ref.
**1**	506 (33,300)	519	0.27 absolute	0.19	10 μM in PBS containing 5% DMSO (V/V)	13.1 μM (HeLa); 7.6 μM (MCF-7)	[6]
**2**	519 (26,300)	533	0.14 absolute	0.14	10 μM in PBS containing 5% DMSO (V/V)	28.6 μM (HeLa); 18.9 μM (MCF-7)	[6]
**3**	504 (68,630)	513	0.03	-	PBS buffer (pH 7.4)	15.7 μM (MCF-7); 19.1 μM (MDA-MB-231); 22.4 μM (SKOV3); 31.0 μM (A2780); 37.0 μM (HeLa); 60.0 μM (A549)	[7]
**4**	596	615	0.04	-	DMSO (10%)-DME; *λ*_ex_ 580 nm	6.1 μM (HeLa); 4.1 μM (A549)	[8]
**5**	710	718	-	-	DMSO (10%)-DME; *λ*_ex_ 685 nm	0.85 μM (HeLa); 1.1 μM (A549)	[8]
**6**	530 (18,000); 500 (27,000)	510	0.09		1% DMSO/DMEM	0.15 μM (HaCaT); 6.7 μM (MCF-7)	[9]
**7**	540 (14,000); 510 (19,000)	-	-	-	1% DMSO/DMEM	0.10 μM (HaCaT); 2.6 μM (MCF-7)	[9]
**8**	505 (41,000); 475 (11,000)	530	0.07	0.26	1% DMSO/DMEM; singlet oxygen quantum yield in air-saturated DMSO/DMF	1.70 μM (A549); 0.4 μM (MCF-7); 1.0 μM (HaCaT)	[10]
**9**	525 (20,100); 490 (7000sh)	540	0.02	0.48	1% DMSO/DMEM; singlet oxygen quantum yield in air-saturated DMSO/DMF	0.09 μM (A549); 0.13 μM (MCF-7); 0.09 μM (HaCaT)	[10]
**10**	555 (11,000)	-	-	0.65	1% DMSO/DMEM; singlet oxygen quantum yield in air-saturated DMSO/DMF	0.05 μM (A549); 0.07 μM (MCF-7); 0.06 μM (HaCaT)	[10]
**11**	650–660	668	0.02	-	*λ*_ex_ 650 nm; 10% DMSO−DMEM of pH 7.2	0.6 μM (HeLa) 0.7 μM (A549) 3.4 μM (MDA-MB-231)	[11]
**12**	617 (29,000)	720	0.032	0.48	10% DMSO/DMEM buffer	1.6 μM (A549) 2.4 μM (HeLa)	[12]
**13**	695 (64,000)	720	0.11	-	H_2_O/1% SDS	~200 μM (A2780P and A2780cisR)	[13]
**14**	~556	582	0.595	-	DMSO	6.41 μM (HeLa)	[14]
**15**	~556	581	0.719	-	DMSO	2.11 μM (HeLa)	[14]
